# Probing intermediates of the induction period prior to nucleation and growth of semiconductor quantum dots

**DOI:** 10.1038/ncomms15467

**Published:** 2017-06-05

**Authors:** Mingyang Liu, Kun Wang, Linxi Wang, Shuo Han, Hongsong Fan, Nelson Rowell, John A. Ripmeester, Romain Renoud, Fenggang Bian, Jianrong Zeng, Kui Yu

**Affiliations:** 1 Institute of Atomic and Molecular Physics, Sichuan University, Sichuan 610065, China; 2 Engineering Research Center in Biomaterials, Sichuan University, Sichuan 610065, China; 3 National Research Council of Canada, Ottawa, Ontario, Canada K1A 0R6; 4 Shanghai Institute of Applied Physics, Chinese Academy of Sciences, Shanghai 201800, China; 5 School of Chemical Engineering, Sichuan University, Sichuan 610065, China

## Abstract

Little is known about the induction period before the nucleation and growth of colloidal semiconductor quantum dots. Here, we introduce an approach that allows us to probe intermediates present in the induction period. We show that this induction period itself exhibits distinct stages with the evolution of the intermediates, first without and then with the formation of covalent bonds between metal cations and chalcogenide anions. The intermediates are optically invisible in toluene, while the covalent-bonded intermediates become visible as magic-size clusters when a primary amine is added. Such evolution of magic-size clusters provides indirect but compelling evidence for the presence of the intermediates in the induction period and supports the multi-step nucleation model. Our study reveals that magic-size clusters could be readily engineered in a single-size form, and suggests that the existence of the intermediates during the growth of conventional quantum dots results in low product yield.

## Abstract (web summary)

Growth of colloidal semiconductor quantum dots involves several stages, including an enigmatic induction period preceding nucleation. Here, the authors identify two distinct intermediates in the induction period, mapping their evolution from precursors all the way to either magic-size clusters or quantum dots.

Colloidal semiconductor nanocrystal (NC) quantum dots (QDs) have recently attracted much attention for their potential applications in biomedical, energy, environmental and security technology fields^1^,^2^,^3^,^4^,^5^,^6^,^7^,^8^,^9^,^10^,^11^. Significant efforts have been focused on the control of size and size distribution, making it possible from a synthetic batch to produce one single nanometer-scale NC ensemble with well-defined electronic and optical properties^12^,^13^,^14^,^15^,^16^,^17^,^18^. In particular, the cadmium chalcogenide (CdE) QD series has been studied widely, serving as model systems for advancing our understanding of the formation mechanisms of NCs^19^,^20^,^21^,^22^,^23^,^24^,^25^,^26^.

The progression from the chalcogen (E) precursors to the final E-based QDs is understood to undergo several developmental stages, including the initial formation of monomers^19^,^20^,^21^,^22^,^23^,^24^,^25^,^26^,^27^,^28^. For example, it has been verified experimentally that in the early stages of the formation of CdSe QDs, the selenide precursor tri-*n*-octylphosphine selenide (TOPSe) is consumed even before nucleation and growth of the QDs^22^; this early stage before nucleation/growth was referred to as the induction period^22^. It is reasonable to assume that during the induction period, the consumption of the precursors produces monomers (Cd_2_E_2_ with a four membered ring stabilized by ligands)^26^, followed by the combination of such monomers into larger cluster-like species^21^. However, regarding the processes that occur during the induction period, there are only a limited number of studies that pertain to the underlying molecular mechanism^20^,^22^,^23^,^24^,^25^,^26^,^27^,^28^. Until now, information on the type of intermediates formed after the formation of monomers (Cd_2_Se_2_) and before nucleation has not been available, and their evolution and relation to the final QD products remain unknown. Understanding of such fundamental details is crucial to fine-tuning the future design and production of high quality QDs that will enable the realization of their potential^1^,^2^,^3^,^4^,^5^,^6^,^7^,^8^,^9^,^10^,^11^.

Usually, a conventional CdE QD ensemble produced in a single reaction batch exhibits one bandgap absorption peak, which is broadened due to the unavoidable presence of size variation that leads to inhomogeneous spectral line broadening^29^,^30^. On the other hand, a CdE magic-size cluster (MSC) ensemble shows a sharper absorption band, with little inhomogeneous spectral line broadening since there is little size variation^12^,^29^,^30^. In fact, owing to their well-defined molecular structures and discrete sizes in the range of 1–2 nm, the MSCs exhibit an absorption peak at a characteristic and consistent wavelength. To distinguish MSCs from conventional QDs, the latter will be referred to as regular QDs (RQDs) in the present study.

A number of synthetic approaches have been documented that typically involved a laborious trial-and-error scheme to produce colloidal semiconductor CdTe (refs 31, 32, 33, 34), CdSe (refs 34, 35, 36, 37, 38, 39, 40, 41, 42), CdS (refs 43, 44) and other composition MSCs^45^,^46^,^47^,^48^. These studies did not address the formation pathway of the MSCs, although some did propose that the MSCs were the actual nuclei for the growth of NCs^13^,^38^,^43^,^47^. However, the coexistence of MSCs of several sizes (instead of a single size) with a single CdE RQD ensemble in one reaction batch does not support such an assumption^34^,^46^. In addition to the synthetic procedures, there is limited information on MSCs obtained from mass measurements^35^,^41^,^48^, energy calculations^35^ and structural characterizations by X-ray diffraction^42^. Even with these investigations, in hand there remains much to discuss about the results^35^,^42^. For example, the energy calculations proposed core-cage structures with 1Cd-to-1Se stoichiometry^35^, whereas the X-ray diffraction measurements suggested tetrahedral structures with off 1Cd-to-1Se stoichiometry^42^. The uncertainty regarding the structure and composition of the CdSe MSCs, which exhibit absorption peaks at 415 nm (ref. 35) or 408 nm (ref. 42), remains an issue to be resolved; critically, the MSCs produced may not be single-sized with the absorption peak positions affected by the presence of other-sized MSCs^35^,^42^. Understanding of the formation pathway of MSCs is essential to advance the production of high quality MSCs in a single-size form, without the presence of other-size NCs, for future structural characterizations.

The study presented here provides the experimental evidence of the presence of intermediates during the induction period before nucleation and growth of colloidal semiconductor NCs. We postulate that, as illustrated in Fig. 1, there is a three-step process which leads to the formation of either MSCs or RQDs. First, the interaction between the Cd and Te precursors at room temperature (RT) results in the formation of supramolecular assemblies of ∼1 nm in an averaged size, inside which the Cd and Te precursors are held together by noncovalent interactions. We denote such a ‘supramolecular-like' or ‘micellar-like' aggregate as **Intermediate 1**. The existence of **Intermediate 1** before the formation of Cd—Te covalent bonds and the simplest covalent species, monomers (Cd_2_Te_2_)^26^, was not expected. Second, Cd—Te bonds form at elevated temperatures such as 130 °C, yielding **Intermediate 2**. The size of **Intermediate 2** is similar to that of **Intermediate 1**. Third, **Intermediate 2** converts to MSCs via intra-molecular re-organization in a beneficial dispersion at room temperature, or, with a continuous supply of thermal energy, RQDs form but not directly from **Intermediate 2**. The evolution of **Intermediate 2** from **Intermediate 1** and the conversion to single-size CdTe MSC-371 (denoted this way in reference to their absorption peak at 371 nm) support a multi-step mechanism of nucleation^49^,^50^,^51^,^52^,^53^,^54^,^55^,^56^. Regarding the existence of **Intermediates 1** and **2** in the induction period, we summarize our characterization in Table 1. The experimental investigation includes the absorption spectroscopy (ultraviolet), synchrotron-based time-resolved *in-situ* and *ex-situ* small angle X-ray scattering (SAXS), electrospray ionization mass spectroscopy (ESI-MS) and ^113^Cd and ^31^P nuclear magnetic resonance (NMR) spectroscopy. Our study offers new insights into the formation of MSCs and RQDs, and application of this new knowledge should enable advances in the production of high-quality MSCs in a single-size form and high-quality RQDs with enhanced particle yield.

## Results

### Evolution of CdTe MSC-371

The synthesis of RQDs has usually been carried out at temperatures where the induction period before nucleation and growth remains very short^12^. Consequently, there has been little investigation focusing on the species evolved in the induction period^22^. With this in mind, we performed reactions at 130 or 135 °C for a relatively long or short induction period, respectively. To explore the species that evolved in the induction period and their possible presence after nucleation/growth of RQDs, the Cd and Te precursors (as described in Methods) were mixed at 135 °C; the reaction mixture was kept at this temperature for a variable length of reaction time from 1 to 90 min. During the first 20 min, the mixture exhibited little colour change, but after 30 min, the colour of the reaction mixture changed rapidly from light green to red, indicating the occurrence of nucleation and growth of RQDs.

In total, nine samples were taken from the synthetic batch kept at 135 °C (Supplementary Figs 1−2). Figure 2 shows the absorption spectra of the first six samples collected at the reaction times up to 50 min. An aliquot (30 μl) of each of the six samples was dispersed in 3 ml toluene (blue traces). When monitoring the nucleation and growth of NCs with absorption spectroscopy, we used toluene to disperse the samples as it has been widely used elsewhere^31^,^32^,^33^,^34^. The absorption spectra obtained from samples (a) to (c) in toluene were relatively featureless, in the detection range of 300–700 nm. For samples (d) to (f) in toluene, we observed the presence of RQDs with broad absorption peaks in the wavelength range of 500–600 nm. Thus, it can be concluded that nucleation/growth of RQDs did not take place in the first 20 min; the reaction period of the first 20 min was the induction period for the formation of RQDs at 135 °C.

To probe the induction period further, we explored various methods (such as those shown in Supplementary Figs 3−4) to identify probable intermediates produced in this reaction period. Surprisingly, it was observed that when an aliquot (30 μl) of each of the aforementioned six samples was dispersed in a 3 ml mixture of octylamine (OTA) and toluene with a volume ratio of 1–5 (red traces in Fig. 2), all except the 1 min sample exhibited sharp absorptions peaked at 371 nm with an increase in optical density (OD) for times up to 20 min. The 1 min sample still gave a featureless absorption spectrum (red), which is similar to that collected in toluene (blue). At the same time, each of the absorption spectra obtained from samples (d) to (f) in amine/toluene overlapped well in the wavelength range of 400–700 nm with the corresponding trace collected from the sample dispersed in only toluene. Interestingly, the absorption spectra of RQDs in the two different dispersions changed little^57^.

The absorption that exhibits a peak at 371 nm with a full width at half maximum (FWHM) as narrow as ∼16 nm suggests the presence of one type of MSCs, which we denote as ‘MSC-371' according to its absorption peak wavelength in nanometres (nm). The appearance of CdTe MSC-371 from one sample only in the amine and toluene mixture (red traces) but not in toluene (blue traces) strongly implies the existence of a particular species in the induction period at 135 °C, which was optically undetectable in conventional toluene. We label such species as ‘intermediates'. Interestingly, when the reaction mixture was heated at 135 °C for only 1 min, the sample in the amine/toluene mixture (red) did not result in the observation of MSC-371. Thus, the intermediate formed at 1 min was not mature enough to result in the formation of MSC-371. Similarly, when the Cd and Te precursors were mixed at room temperature and held for one day, the intermediate that could result in MSC-371 was not formed either. Accordingly, when this Cd and Te mixture was dispersed into the amine mixture, featureless absorption was detected, as shown in Supplementary Fig. 4.

To differentiate the two types of the intermediates, we denote **Intermediate 1** and **Intermediate 2** to represent those which do not and which do lead to MSC-371 in an amine/toluene mixture, respectively. The results in Fig. 2, together with those in Supplementary Figs 1−4, collectively indicate that the formation of **Intermediate 2** is necessary for the observation of MSC-371 in the amine/toluene mixture. We now focus on the characterization of **Intermediates 1** and **2**, mainly. The sample preparation condition can be found in Supplementary Fig. 4, and the induction period for the reaction at 130 °C lasted at least up to 30 min.

### SAXS study of the intermediates and MSCs formed

**Figure 1:**
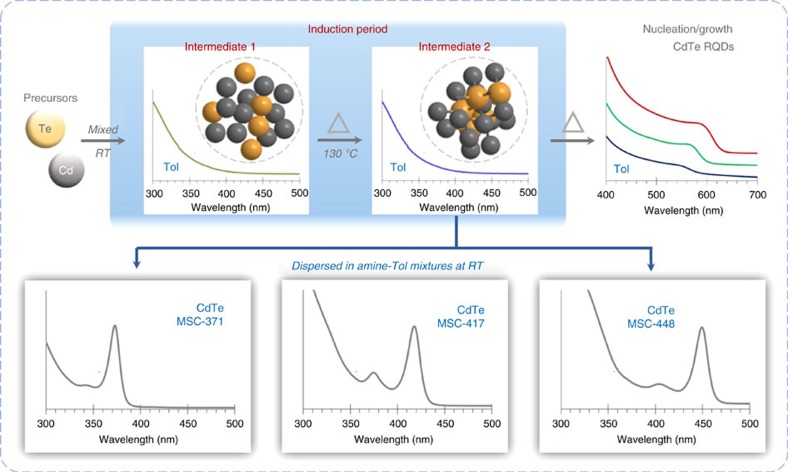
Schematic illustrating the formation pathways of intermediates, magic-sized clusters, and quantum dots. In traditional synthetic batches leading to colloidal regular quantum dots (RQDs which exhibit bandgap absorption in toluene that redshifts as the size increases), there is a so-called induction period occurring before QD nucleation and growth. With CdTe, during the induction period the development of **Intermediate 1** at room temperature (RT) is followed by the formation of **Intermediate 2** at elevated temperatures. Both intermediates are ∼1 nm in size, and their properties were monitored by optical absorption spectroscopy, SAXS, MS and NMR. Before monomers are formed, the interaction between the Cd and Te precursors at RT leads to the formation of **Intermediate 1**. With an increase in the reaction temperature, Cd—Te covalent bonds form, leading to **Intermediate 2**, after which maintaining a continuous supply of thermal energy causes RQDs to form. These two types of intermediates exhibit featureless absorption in toluene (Tol). However, when **Intermediate 2** is dispersed in mixtures of a primary amine and toluene at RT, CdTe MSCs are formed, namely MSC-371, MSC-417, and MSC-448, each as a sole product without the coexistence of NCs of other sizes.

Small angle X-ray scattering (SAXS) has been widely used to study structural information of nanoscale or even microscale systems of particles. The small angular range of X-ray scattering can deliver information including the size, size distribution and morphology of targets of interest^58^,^59^. We used synchrotron-based *in-situ* and *ex-situ* SAXS to investigate the intermediates and MSCs. We considered the reaction between the Cd and Te precursors, as a process of the formation of particles in a two-electron-density system, with one corresponding to the particle as solute and the other as solvent^59^,^60^,^61^,^62^. Figure 3a shows the *in-situ* SAXS data of the reaction mixture of the Cd and Te precursors mixed at room temperature (30 °C) and heated (with the rate of 20 °C per minute) up to 150 °C. The 30, 90 and 150 °C data are presented. Figure 3b presents the *ex-situ* SAXS data of the 130 °C/30 min sample collected at room temperature and the developed MSC-371, MSC-417 and MSC-448. The absorption spectra of the three types of MSCs are presented in Fig. 1. The unified model proposed by Beaucage has been documented to calculate practical sizes and morphologies of multi-size-scale structures in one sample based on scattering over a wide range of the scattering vector *q* (refs 59, 60, 61, 62); thus, we applied this model to analyse the data. The corresponding fitting with the unified model is shown in Fig. 3 as the solid curves (with corresponding colours used for the experimental data). The detailed information on data analysis is presented in the Methods.

For the reaction mixture heated from 30 °C to 90 °C and 150 °C, the scattering data suggest the presence of particulate intermediates, which are monodispersed in the solvent with the size increasing from 1.05 nm to 1.28 nm and 1.50 nm, respectively. Here, the word ‘size' represents the overall diameter. At this stage, noted as L1, there is no additional assembly of the primary cluster species leading to intricate hierarchical architectures. A significant and novel observation is the 1.05 nm species, which were detected after the Cd and Te precursors were mixed at 30 °C, with scattering correlation distance *ξ*=2.32 nm. Interestingly, such a behaviour of aggregation or self-assembly at room temperature takes place before the formation of Cd—Te covalent bonds, as suggested by our ESI-MS (Fig. 4 and Supplementary Fig. 5) and NMR (Fig. 5 and Supplementary Figs 8–10). Thus, **Intermediate 1** at room temperature is ∼1 nm in size and is ‘supramolecular-like', consisting of the Cd and Te precursors held together by noncovalent interactions, free of Cd—Te covalent bonds.

Along with the increase of temperature, these intermediate precursors became slightly larger from 1.05 nm at 30 °C to 1.28 nm at 90 °C and 1.50 nm at 150 °C. Also, the scattering correlation distance *ξ* decreased somewhat from 2.32 nm at 30 °C to 2.28 nm at 90 °C and 2.17 nm at 150 °C. This decrease in *ξ* was accompanied by a decrease in the pack factor *k* (typically in the range of 0–6) from 2.06 at 30 °C to 1.80 at 90 °C and 1.39 at 150 °C (Supplementary Table 2). The decrease of *ξ* along the temperature increase might be related to the assembly tendency of the intermediate precursor species. The decrease of *k* contributed mainly to the increase of the scattering intensity at the small *q* range.

For the 130 °C/30 min mixture (with the presence of **Intermediate 2**) in toluene, its *ex-situ* scattering pattern obtained at room temperature shown in Fig. 3b is quite similar to those shown in Fig. 3a. Clusters of 0.97 nm in size were obtained from the fitted model shown by the grey line (Fig. 3b). Thus, the size and morphology of **Intermediate 2** formed did not change much when the mixture temperature was cooled down from 130 °C to room temperature. For MSC-371, MSC-417 and MSC-448 developed in dispersions, the scattering data collected at room temperature (Fig. 4b) suggest the presence of two levels of structures. The primary structure, labelled as L1, has the diameter of 0.97, 1.39 and 1.65 nm, respectively. The secondary structure, addressed as L2, which is likely formed via the self-assembly of the primary species, is thus much larger with an overall diameter of 19.48, 21.77 and 24.24 nm, respectively. It seems that the mass fractal of the secondary structures sits somewhere between a collapsed and swollen polymeric aggregation^63^. L2 is indicative of a ready assembly of these MSCs. Interestingly, **Intermediate 2** (formed in the 130 °C/30 min reaction mixture) dispersed in toluene at room temperature and MSC-371 (formed from this 0.5 ml dispersion diluted with 0.5 ml OTA after 10 min) both gave a size of 0.97 nm when fitted. Importantly, the SAXS patterns of the 130 °C/30 min and MSC-371 samples are obviously different. Thus, our SAXS study seems consistent with our absorption results, which suggest that the formation of MSC-371 does not take place at 135 °C but in the amine-toluene mixture at RT. For this reason, we can hypothesize that the absence of MSC-371 in toluene is not due to dissolution.

### Mass spectroscopy study of the intermediates

Electrospray ionization mass spectroscopy (ESI-MS) has been documented to characterize organometallic nanoclusters, with a very much limited body of literature on semiconductor QDs^41^,^48^,^64^. ESI-MS was employed here to study the intermediates formed in the induction period of colloidal semiconductor QDs. Figure 4a shows two high resolution ESI-MS spectra, which were obtained via the positive-ion mode within the *m*/*z* range from 900 to 1,500 from two reaction mixtures at room temperature for 24 h (spectrum 1) and at 130 °C for 30 min (spectrum 2). When the Cd and Te precursors were mixed at room temperature for 24 h, there was no fragment of Cd_*x*_Te_*y*_ detected within the *m*/*z* range from 900 to 1,500 (spectrum 1 of Fig. 4a, and spectrum 1 of Supplementary Fig. 5 within the 800 to 1,500 *m*/*z* range). Accordingly, there was no formation of Cd—Te covalent bonds for **Intermediate 1,** which is formed at room temperature, correlating with the fact that no MSC-371 was detected in the amine-toluene mixture (Supplementary Fig. 4).

When the Cd and Te precursors were mixed at 130 °C and kept at this temperature, interestingly, fragments of Cd_*x*_Te_*y*_ consisting of Cd and Te isotopic peaks were detected from the 130 °C samples with different reaction times (Fig. 4 and Supplementary Figs 5−6). Five groups of fragments were observed; the relatively dominant peaks of each of the five groups are labelled with star symbols at *m*/*z* 929, 1,041, 1,169, 1,316 and 1,428. From the Cd and Te isotopic patterns and peak positions (Supplementary Fig. 7), we were able to match the experimental mass fragments with calculated weights of Cd_*x*_Te_*y*_. These five fragments are thus assigned to Cd_6_Te_2_, Cd_7_Te_2_, Cd_7_Te_3_, Cd_6_Te_5_ and Cd_7_Te_5_, respectively. Closer examination reveals that from left to right, the fragments consisting of Cd_*x*_Te_*y*_ isotopes are attributed to Cd_7_Te_1_, Cd_6_Te_2_ and Cd_5_Te_3_ (Fig. 3b), Cd_8_Te_1_, Cd_7_Te_2_, and Cd_6_Te_3_ (Fig. 3c), Cd_7_Te_3_ and Cd_5_Te_5_ (Fig. 3d), Cd_7_Te_4_, Cd_6_Te_5_, and Cd_5_Te_6_ (Fig. 3e), and Cd_7_Te_5_ and Cd_6_Te_6_ (Fig. 3f). These positively charged fragments, which were stripped off from the intermediate by electrospray ionization, have a general formula of Cd_*x*_Te_*y*_, with *x*+*y*=8 (b), 9 (c), 10 (d), 11 (e) and 12 (f). Density functional theory (DFT) calculations will be performed in the future to elaborate the probable skeleton of the Cd_*x*_Te_*y*_ fragments. Two candidate skeletons of the Cd_6_Te_2_ and Cd_7_Te_2_ fragments are suggested in Fig. 3g,h, respectively. With the formation of Cd—Te covalent bonds, **Intermediate 2** formed in the 130 °C/30 min sample resulted in the formation of MSC-371 in the OTA and toluene mixture (Supplementary Fig. 4). Again, **Intermediate 2** with the formation of Cd—Te covalent bonds is optically invisible in toluene and becomes optically visible in the OTA and toluene mixture via the expression of itself as MSCs.

It is generally accepted that the physicochemical property of a sample affects ionization efficiencies^41^,^48^. Here, our surface amine ligands could readily be detached by the ionization process of ESI, due to their low-dissociation energy from the surface atoms. Hence, the fragments detected by ESI-MS describe the bare Cd_*x*_Te_*y*_ clusters without surface ligands; basically, the fragments were 7–12 atom structured with the expected uncertainty (Supplementary Fig. 6), regarding the matching of the experimental observation with calculated weight due to the very wide isotope distributions^64^. The detection of the Cd_*x*_Te_*y*_ fragments from the 130 °C/30 min sample provides evidence for the evolution of **Intermediate 2** consisting of Cd—Te bonds. The absence and presence of the Cd—Te bond in the reaction mixture at room temperature for **Intermediate 1** and at 130 °C/30 min for **Intermediate 2**, respectively, is in consistent with our SAXS (Fig. 3) and NMR (Fig. 5) measurements reported in the above and following two sections.

### NMR study of the intermediates

With sensitivity to local environment of nuclides such as ^31^P and ^113^Cd, nuclear magnetic resonance (NMR) spectroscopy has been used to provide valuable information on the bond formation between metal (M) and chalcogenide (E), which leads to the M_2_E_*n*_ monomers of semiconductor M_2_E_*n*_ NCs^20^,^22^,^23^,^24^,^25^,^26^,^27^,^28^, and on the structure and composition of M_2_E_*n*_ NCs of interest^15^,^16^,^18^,^44^,^45^,^46^,^47^,^65^. Here, we used ^113^Cd and ^31^P NMR spectroscopy to study the formation of **Intermediates 1** and **2** in the Cd and Te precursor mixtures without purification. H_3_PO_4_ and Cd(ClO_4_)_2_ were used as the chemical shift references, respectively. First of all, reference ^31^P NMR spectra of TOP (trace 1) and TOPTe (trace 2) are presented in Fig. 5a and Supplementary Fig. 8. Meanwhile, reference ^113^Cd NMR spectrum of the Cd precursor Cd(OAc)_2_(OLA)_*x*_ used (trace 3) is presented in Fig. 5b and Supplementary Fig. 9. Figure 5 then presents the ^31^P (a) and ^113^Cd (b) NMR spectra collected at room temperature from five reaction mixtures of the Cd and Te precursors, held at different reaction temperatures and periods: at RT for 24 h (4), at 130 °C for 1 min (5), 10 min (6), 30 min (7), 90 min (8). The absorption spectra of the 1 min, 30 min and 90 min samples at 130 °C are shown in Supplementary Fig. 4; the presence of NCs was detected for the 90 min sample.

The ^31^P resonance signal of our Te precursor TOPTe (made with the feed molar ratio of 4TOP-to-1Te) was at −27.2 p.p.m. (spectrum 2 of both Fig. 5a and Supplementary Fig. 8). It was previously reported that the TOP-to-Te feed molar ratios affect the ^31^P resonance signal; the more TOP, the greater the up-field shift. When the feed molar ratio was 1-to-1, 3-to-1, and 6-to-1, the ^31^P resonance signal was located at −14.1 p.p.m., −25.3 p.p.m. and −28.6 p.p.m., respectively^26^. Thus, the presence of one single broad peak instead of two, one for TOP and the other for TOPTe, may be due to chemical exchange between free TOP and bound forms, similar to that collected from mixtures of cadmium oleate (Cd(OA)_2_) and TOP^23^. The ^113^Cd resonance signal of our Cd precursor Cd(OAc)_2_(OLA)_*x*_ (made with the feed molar ratio of 1Cd(OAc)_2_-to-12OLA) was at 137.0 p.p.m. (spectrum 3 of both Fig. 5b and Supplementary Fig. 9). Also, the ^113^Cd resonance signal strongly depends on the feed Cd(OAc)_2_-to-OLA molar ratios (Supplementary Fig. 9). It is observed that the more OLA is used, the greater down-field shift is observed. When the feed molar ratio was 1-to-3, 1-to-6, and 1-to-12, the ^113^Cd resonance signal was situated at 87.0 p.p.m., 110.0 p.p.m. and 137.0 p.p.m., respectively. Such an observation may be due to the chemical exchange as well.

After the Cd and Te precursors were mixed at room temperature for 24 h, the ^31^P resonance signal shifted down-field to −26.5 p.p.m. (spectrum 4 of both Fig. 5a and Supplementary Fig. 8), while the ^113^Cd resonance signal did not exhibit a noticeable shift and was located at 137.2 p.p.m. (spectrum 4 of both Fig. 5b and Supplementary Fig. 9). The coordination of TOP or TOPTe to Cd was reported to result in down-field shift of the ^31^P resonance signal^23^,^24^,^25^,^26^. The ^113^Cd resonance signal at 137.2 p.p.m. seemed to be dictated mainly by the interaction with the relatively large amount of OLA instead of the relatively small amount of TOPTe. The down-field shift of the ^31^P resonance signal and trivial change of the ^113^Cd resonance signal should be caused by the probable interaction between the Cd and Te precursors. It is clear as in the absorption study shown in Supplementary Fig. 4, the interaction was weak and did not lead to nucleation or growth of NCs. Meanwhile, such a weak interaction led to the presence of **Intermediate 1** with the size of ∼1 nm as suggested by our SAXS study (Fig. 3a) and ESI-MS study (Fig. 4a). This result is also in agreement with our preliminary study using diffusion ordered spectroscopy (DOSY) NMR (Supplementary Fig. 10) which monitored the decrease of the diffusion coefficient of the P-containing species (TOPTe) after the presence of the Cd precursor.

After the Cd and Te precursors were mixed at 130 °C, nucleation and growth of RQDs did not occur in the first 30 min, as demonstrated by the corresponding absorption spectra shown in Supplementary Fig. 4. The ^31^P resonance signal exhibited an up-field shift from −27.4 p.p.m. at 1 min (Fig. 5a, spectrum 5), −27.9 p.p.m. at 10 min (6), to −28.4 p.p.m. at 30 min (7). The release of free TOP could result in the up-field shift observed^23^. Again, single broad peaks were obtained, instead of the appearance of a sharp^31^ P peak (as in the reference spectrum of TOP Fig. 5a, spectrum 1), a TOPTe peak and the other peaks including a TOP−Cd peak. The occurrence of a single broad peak could be again resulted from the chemical exchange between free TOP and these bound forms. At the same time, the ^113^Cd resonance signal broadened and shifted in the down-field direction from 137.3 p.p.m. at 1 min (Fig. 5b, spectrum 5), 144.4 p.p.m. at 10 min (6), to 156.8 p.p.m. at 30 min (7).

Our previous solid-state NMR studies have found that the core Cd atoms of a QD appear more down-field than those surface ones^18^,^44^,^45^. For example, with Cd(NO_3_)_2_·4H_2_O (powder) as a chemical shift reference for ^113^Cd solid-state NMR, the core Cd (bounded to Te only) of CdTe MSC-428 (capped by carboxylic acids) was found to be at the down-field side (485 p.p.m.), while the surface Cd (bounded to Te and surface ligand –COO^−^) was at the up-field side (357 p.p.m.)^45^. Correspondingly, the two core and surface ^113^Cd resonance signals should be at around 385 p.p.m. and 257 p.p.m., respectively, if Cd(ClO_4_)_2_ were a reference (Supplementary Table 1). Thus, the present down-field shift of the ^113^Cd resonance signal from 137.3 p.p.m. (1 min sample) to 156.8 p.p.m. (30 min sample) during the induction period at 130 °C signifies the probable formation of Cd—Te bonds. In solution there is not only coordination of Cd to Te, but also to OLA and TOP, and this is likely responsible for the chemical shift difference between the solid and solution. Note that the Cd species is in excess, as compared to the Te species, and no purification was carried out. Again, it is the chemical exchange that could be responsible for the presence of a single broad ^113^Cd resonance peak. It is known that the relaxation time *T*_2_ is inversely related to linewidth (FWHM). Thus, the broadening indicates that *T*_2_ was reduced due to possible contributions from the chemical exchange involving a number of related species, and also from the formation of larger aggregates or micelles that exhibit slower motion^66^. The formation of **Intermediates 1** and **2** with the size of ∼1 nm is thus expected to be contributing to the broadening as well. Accordingly, ^31^P and ^113^Cd NMR in Fig. 5 is in agreement with the formation of **Intermediate 1** at room temperature, followed by **Intermediate 2** with the bond formation between Cd and Te at 130 °C, which is accompanied by the release of free TOP^23^.

After around 90 min at 130 °C, nucleation and growth was observed (as shown in Supplementary Fig. 4). Meanwhile, the ^31^P (Fig. 5a, spectrum 8) and ^113^Cd (Fig. 5b, spectrum 8) resonance signals sharpened and shifted to the up-field direction at −32.1 p.p.m. and 142.0 p.p.m., respectively. The ^31^P signal change is apparently related to the further release of TOP. The ^113^Cd signal change is presumably due to a rearrangement of the location and coordination of Cd atoms. This observation is in agreement with the fact that the surface of NCs is Cd-rich^18^,^67^. As mentioned above, the resonance signal of surface Cd is located on the up-field side, when compared to the signal for the core or interior Cd of a QD^18^,^44^,^45^. Here, the Cd atoms at the surface bind both to surface ligands and to Te, while the interior Cd atoms bind only to Te. Once more, it may be the chemical exchange that leads to the presence of one ^113^Cd resonance signal, even with the presence of un-reacted Cd precursors. It is noteworthy that it is possible to distinguish, from the ^113^Cd resonance signals obtained, the Cd and Te precursors used (Fig. 5b, spectrum 3), the supramolecular assembly of **Intermediate 1** at the very beginning stage of the induction period (Fig. 5b, spectrum 4), **Intermediate 2** formed at the later stage of the induction period (Fig. 5b, spectrum 7), and the RQDs formed (Fig. 5b, spectrum 8).

These NMR results comprehensively reveal the changes of the ^113^Cd and ^31^P resonance signals in the forward reaction, all the way from the Cd and Te precursors to **Intermediate 1** and then to **Intermediate 2** generated in the induction period, and furthermore to the RQDs produced after nucleation and growth. In general, the combination of our experimental results presented in Figs 3, 4, 5 provides experimental support for the existence of **Intermediates 1** and **2** with the overall diameter of ∼1 nm. At room temperature, noncovalent Cd and Te interaction leads to the presence of the **Intermediate 1** which is ‘supramolecular-like' without the formation of Cd—Te bonds. Along the increase of reaction temperature, the formation of Cd—Te bonds takes place, leading to the presence of **Intermediate 2**.

## Discussion

In the study presented here, we show that we have effectively decoupled the complex reaction stages and achieved the stabilization of two **Intermediates**, denoted as **1** and **2**, which we hypothesize to be formed during the induction period before the nucleation and growth of RQDs. Our observations are summarized in Table 1. When the Cd and Te precursors were mixed at room temperature, we detected ∼1 nm species by SAXS, which we hypothesize reflects the formation of **Intermediate 1**. At this stage, it appears that Cd—Te covalent bonds have not yet formed, as suggested by our ESI-MS and NMR measurements. Thus, the Cd and Te precursors that constitute **Intermediate 1** are likely held together by noncovalent interactions. The existence of such ‘supramolecular-like' aggregates before the formation of covalently bonded monomers (Cd_2_Te_2_)^26^ was quite unexpected. The ^113^Cd/^31^P NMR resonances were observed to change from 137.0 p.p.m./−27.2 p.p.m. for the Cd/Te precursors, to 137.2 p.p.m./−26.5 p.p.m. for their mixture held at room temperature for 24 h (**Intermediate 1**), then to 137.3 p.p.m./−27.4 p.p.m. for the mixture kept at 130 °C for 1 min, and finally to 156.8 p.p.m./−28.4 p.p.m. for the mixture kept at 130 °C for 30 min (**Intermediate 2**). The continuous down-field shift and broadening of the ^113^Cd NMR signal, together with the general up-field shift of the ^31^P NMR signal, may be explained by progressive formation of Cd—Te covalent bonds, thus supporting our hypothesis that **Intermediates 1** and **2** gradually evolved during the induction period. ESI-MS detected the fragments of Cd_*x*_Te_*y*_ (with *x*+*y*=7 to 12) from the sample heated at 130 °C for 30 min (**Intermediate 2**), which is also consistent with the formation of Cd—Te covalent bonds. SAXS results suggested that the sizes of **Intermediates 1** and **2** at room temperature were similar at ∼1 nm.

We have compared the optical absorption spectra of **Intermediates 1** and **2** in toluene, together with those of the Cd and Te precursors. These absorption spectra are shown in Supplementary Fig. 11; **Intermediates 1** and **2** were represented by the mixture of the two precursors after two different kinds of treatment, with the former after standing at room temperature for 24 h and the latter after heating at 130 °C for 30 min. It is a reasonable assumption that during the induction period at room temperature, **Intermediate 1** was present along with the individual Cd and Te precursors. After heating at 130 °C/30 min, both **Intermediates 1** and **2** are likely to coexist. Similar to the case for small molecules (such as TOPTe), the absorption peaks of **Intermediates 1** and **2** in toluene should be located at wavelengths shorter than 300 nm. Thus, both **Intermediates 1** and **2** are ‘optically-invisible' in the conventional detection range in toluene, and their absorption spectra in toluene were similar in the detection wavelength range longer than 300 nm.

**Figure 2:**
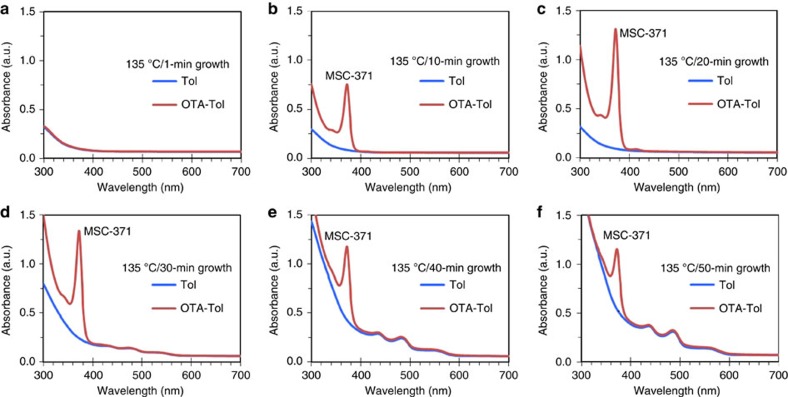
Formation of CdTe MSC-371 in a mixture of amine and toluene but not in toluene. The Cd and Te precursors were mixed at 135 °C and reacted for 1 min (**a**), 10 min (**b**), 20 min (**c**), 30 min (**d**), 40 min (**e**) and 50 min (**f**). An aliquot (30 μl) of each of the six samples was dispersed into 3 ml of toluene (blue) or the mixture of 0.5 ml octylamine (OTA) and 2.5 ml toluene (red). The spectra were collected at room temperature (∼25 °C) after 1 min of dispersion. Interestingly, CdTe MSC-371 was detected in samples **b** to **f** in the mixture of OTA and toluene (red) but not in the toluene (blue) dispersions.

To study the ‘conventionally-invisible' intermediates, we added a primary amine into toluene to disperse our samples before and after the nucleation/growth of RQDs (Fig. 2). The primary alkyl amine has been employed as an additive to engineer various colloidal semiconductor RQDs^24^,^25^. This additive promoted the evolution of the ‘optically-visible' MSCs from ‘invisible' **Intermediate 2**. Thereby, we obtained CdTe MSC-371, MSC-417 and MSC-448 as single-sized species, without the coexistence of other sized NCs. Their sizes were 0.97 nm, 1.39 nm and 1.65 nm, respectively, as determined by SAXS. Thus, it is evident that MSCs can be produced at room temperature when **Intermediate 2** is dispersed in a proper environment. Intriguingly, our preliminary ^1^H NMR experiments (Supplementary Fig. 12) demonstrated that the chemical shift of the proton (which is bonded to the nitrogen atom of a primary amine molecule) is different upon the presence of the Cd source. Since the addition of primary amine molecules into toluene was found to be necessary, a proton such as that provided by the addition of primary amine molecules may play an important role in the formation of the MSCs from **Intermediate 2**. We are actively studying such an effect of acidic protons including the use of alcohols. When thermal energy was supplied continuously, RQDs were generated at elevated temperatures via a conventional nucleation/growth process. The dispersion of **Intermediate 2** in a beneficial environment at room temperature suppresses the nucleation and growth of RQDs, with the very significant advantage that a single-size MSC ensemble can be engineered, as illustrated in Fig. 1. Traditional approaches to synthesize MSCs were at elevated temperatures and involved possible nucleation/growth of RQDs. Thus, the current study proposes a synthetic precedent for MSCs with much improved control via **Intermediate 2** formed during the induction period.

It is apparent that the formation of MSC-371 took place exclusively in the amine-toluene mixture at room temperature and not in the reaction at elevated temperatures such as at 130 °C. The evidence for this conclusion comes from the different SAXS patterns obtained at room temperature from the **Intermediate 2** sample (130 °C/30 min) and from the single-size MSC-371 sample. The use of additives to provide compelling evidence in the study of intermediates produced in reactions has been a common practice employed by organic chemists^68^,^69^,^70^. Evidently, the development of MSC-371 from the Cd and Te precursor mixture via **Intermediate 2** after the initial formation of **Intermediate 1** does not seem to follow the conventional mechanism envisaged by classical nucleation theory (CNT), but agrees well with the multi-step mechanism of nucleation, previously reported for the formation of calcium carbonate and calcium phosphate^49^,^50^,^51^,^52^,^53^,^54^,^55^,^56^. In those earlier studies, the formation of stable pre-nucleation mineral clusters was first suggested by the observation that the number of free calcium ions detected was obviously smaller than that of calcium ions added. It was explained that in an unsaturated solution, calcium ions were bound in some fashion. Accordingly, pre-nucleation clusters were suggested to be stable and essentially liquid-like ionic polymers consisting of alternating calcium and carbonate ions in various forms such as chains, branches, and rings^51^. Furthermore, the present study on the formation of **Intermediate 1** echoes the previous study showing that the Cd source Cd(OAc)_2_ was polymeric like in toluene; in the presence of OTA, monomeric complexes Cd(OAc)_2_(OTA)_*x*_ (*x*=2 or 4) form, which is unlikely to be larger than a dimer^71^. In the presence of TOPTe, **Intermediate 1** forms at RT, as supported by our SAXS and DOSY NMR.

We note that the nucleation and growth of RQDs could well deplete the amount of **Intermediate 2**, as suggested by the results shown in Fig. 2. We see that the amount of MSC-371 increased but only for times up to 20 min; afterwards, the amount of MSC-371 continuously decreased for times up to 90 min (Supplementary Figs 1 and 2). Obviously, the transition from increasing to decreasing the amount of MSC-371 corresponded to the start of the nucleation and growth process of the RQDs, which was detected to be around 30 min. These results provide a strong indication that the amount of MSC-371 detected is correlated to the quantity of **Intermediate 2**. Thus, the variation of the optical density of MSC-371 in the amine mixture seemed to follow the generation and disappearance of **Intermediate 2** in the reaction batch at 135 °C. The generation took place during the induction period, and the disappearance occurred as a result of the nucleation/growth of RQDs. After the induction period, the formation of RQDs was facilitated by a continuous supply of thermal energy. The existence of the previously unrecognized intermediate for RQDs could provide a basis for explaining their chronically low production yield, particularly for small-sized QDs which require a relatively low reaction temperature and/or a relatively short reaction period^23^,^28^.

In conclusion, we have examined the evolution of the intermediates which occur during the induction period before nucleation and growth of colloidal semiconductor compound RQDs. Our characterization methods included a combination of UV absorption spectroscopy, synchrotron-based time-resolved *in-situ* and *ex-situ* SAXS, MS, and ^113^Cd and ^31^P NMR. Owing to our ability to decouple the reaction stages, that is to separate the induction period from nucleation/growth of RQDs, we could engineer three types of CdTe MSCs as a single-sized reaction product with the absence of other-size MSCs and/or RQDs. Our three-step model on the formation of MSCs and RQDs (Fig. 1) provides valuable insights also for the other research fields such as those involving noble metal clusters, noble metal particles and perovskites. Nonetheless, several important issues remain to be resolved, such as the chemistry leading to the formation of **Intermediate 2** after the formation of Cd_2_Te_2_ monomers^26^, their actual formulae and transformation processes which lead to the detected MSCs. We are actively exploring these fundamental aspects related to the very beginning stage before the nucleation and growth of colloidal semiconductor compound RQDs, together with those involved in the formation of MSCs. Also, we will address whether fast exchange of Cd species between MSCs and solution Cd species is likely or unlikely. The present effort, together with studies on the structure and chemical shift of Cd(OAc)_2_ in various solvents^71^,^72^ and mechanistic studies on the formation of monomers^19^,^20^,^21^,^22^,^23^,^24^,^25^,^26^,^27^,^28^, explores the chemistry at the nanoscale, providing much more in-depth understanding of the induction period, which has been largely unexplored but is critically important. Similar to the advance of organic syntheses in the first half of the twentieth century, the field of colloidal semiconductor NCs is moving forward steadily from an empirical art to science.

## Methods

### Chemicals

All chemicals used are commercially available and were used as received (or otherwise specified). Cd and Te sources are Cd(OAc)_2_·2H_2_O (99.999%) and tellurium powder (99.99%, 325 mesh), respectively; they were purchased from Alfa Aesar. The amine used for our reactions is oleylamine (70%, OLA). Tri-*n*-octylphosphine (90%, TOP) was used to make the TOPTe precursor. The amines used for our dispersions include octylamine (99%, OTA). The solvents used include toluene (99%). They were purchased from Sigma Aldrich.

### Sample preparation

Our Cd precursor, Cd(OAc)_2_(OLA)_*x*_, was prepared from cadmium acetate Cd(OAc)_2_ in OLA^39^. To prepare the Cd precursor, Cd(OAc)_2_·2H_2_O (2.35 g, 8.63 mmol) and OLA (15.00 ml, 32.58 mmol) were placed in a three-neck flask. The Cd concentration is 0.573 mmol ml^−1^. The mixture was degassed under vacuum three times at room temperature. Then, the temperature was increased to 120 °C for one hour under vacuum, and a clear light orange solution was obtained. Under a N_2_ atmosphere, the reaction was cooled down to room temperature; the reaction flask was transferred to a glove box.

Our Te precursor was tri-*n*-octylphosphine telluride (TOPTe) prepared with a feed molar ratio of 4TOP to 1Te. To prepare a Te stock solution, Te powder (0.56 g, 4.40 mmol) and TOP (6.52 g, 17.60 mmol) were placed in a three-neck flask; the Te concentration was 0.56 mmol ml^−1^. The mixture was degassed three times at room temperature. Under N_2_, the temperature of the mixture was increased to 350 °C for 30 min after which a clear orange yellow solution was obtained. When the solution was cooled down to room temperature, the colour of the mixture changed to greenish yellow; the flask was transferred into a glove box.

**Figure 3:**
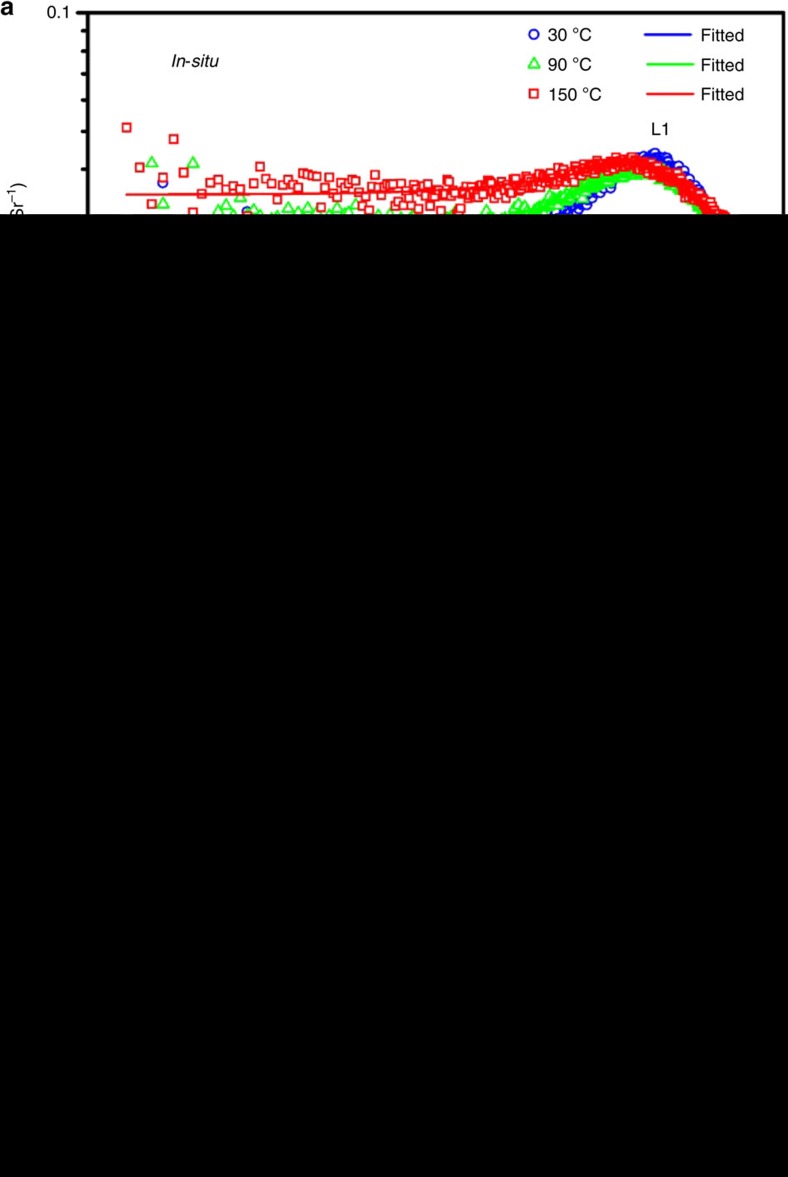
*In-situ* and *ex-situ* SAXS data of intermediates and MSCs. (**a**) *In-situ* profiles showing the evolution of the intermediate in the induction period, with the overall diameter increasing from 1.05 nm at 30 °C (blue circular symbols) to 1.28 nm at 90 °C (green triangular symbols) and 1.54 nm at 150 °C (red squared symbols). Inset shows the model of monodisperse intermediates. (**b**) *Ex-situ* SAXS profiles for the 130 °C/30 min **Intermediate 2** (dispersed in dry toluene with the 1–4 volume ratio of sample to toluene, grey star symbols), MSC-371 (in 0.5 ml OTA for 10 min, blue circular symbols), MSC-417 (in the mixture of 0.2 ml OTA, 0.2 ml ethanol and 0.1 ml toluene for 60 min, green triangular symbols) and MSC-448 (in the mixture of 0.1 ml ethanol and 0.4 ml toluene for 10 min, red square symbols). Each of the three MSC dispersions was prepared from the same 130 °C/30 min sample in toluene shown in Fig. 4b (grey) of 0.5 ml. The diameters of the three types of MSCs (*D*_1_, primary structure) are 0.97, 1.39 and 1.65 nm, respectively. The overall diameters of the MSC aggregation (*D*_2_, secondary structure) are 19.48, 21.77 and 24.24 nm, respectively. Inset shows the model of MSCs and their aggregation. The concentration of the *in-situ* sample is higher than those of the *ex-situ* samples. Details can be found in Methods.

**Figure 4:**
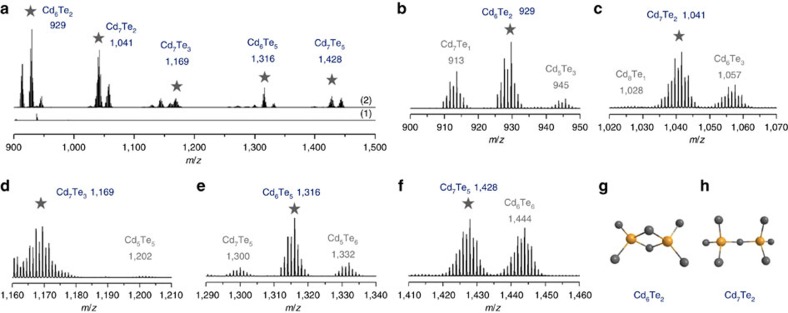
Mass spectrometry of intermediate species. (**a**) ESI-MS spectra of the RT/24 h (1, **Intermediate 1**) and 130 °C/30 min (2, **Intermediate 2**) samples. For the former mixture, fragments in the region of *m*/*z* 900–1,500 were not detected, suggesting that Cd—Te covalent bonds did not form for the **Intermediate 1** stage. For the latter sample, five groups of Cd_*x*_Te_*y*_ fragments were detected, dominated by Cd_6_Te_2_, Cd_7_Te_2_, Cd_7_Te_3_, Cd_6_Te_5_ and Cd_7_Te_5_ (labelled by star symbols at *m*/*z* 929, 1,041, 1,169, 1,316 and 1,428, respectively). Close examination of the five groups of Cd_*x*_Te_*y*_ fragments (*x*+*y*=8 (**b**), 9 (**c**), 10 (**d**), 11 (**e**) and 12 (**f**)) reveals the presence of both Cd and Te isotopes, suggesting the formation of Cd—Te covalent bonds for the **Intermediate 2** stage. Possible skeletons (drawn by ChemOffice) for Cd_6_Te_2_ (**g**) and Cd_7_Te_2_ (**h**) are suggested.

**Figure 5:**
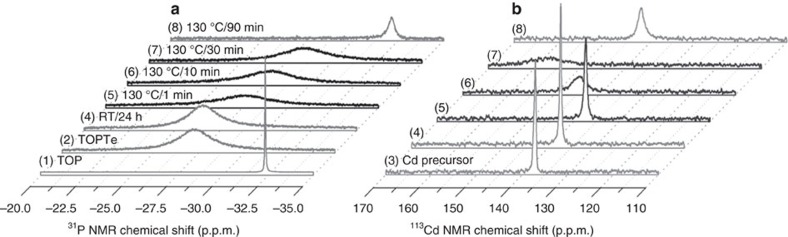
^31^P and ^113^Cd NMR spectra. (**a**) ^31^P {^1^H} NMR investigation of the induction period of the five reactions of Cd(OAc)_2_(OLA)_*x*_+TOPTe (**4**–**8** as indicated), together with reference 90% TOP purchased (**1**, −32.4 p.p.m.) and the Te precursor used TOPTe (**2**, −27.2 p.p.m.). The ^31^P resonance signal is at −26.5 p.p.m. (**4**), −27.4 p.p.m. (**5**), −27.9 p.p.m. (**6**), −28.4 p.p.m. (**7**) and −32.1 p.p.m. (**8**). The ratio of the integral of the peak to that of all the other P-containing species is 16.7 (**4**), 6.7 (**5**), 5.8 (**6**), 4.3 (**7**) and 0.8 (**8**) (Supplementary Fig. 8). Obviously, the consumption of TOPTe takes place before the formation of NCs (which were detected in Sample **8** only). (**b**) ^113^Cd NMR investigation of the five reactions of (**4**–**8**), together with the Cd precursor used (**3**). The ^113^Cd resonance signal is at 137.0 p.p.m. (**3**), 137.2 p.p.m. (**4**), 137.3 p.p.m. (**5**), 144.4 p.p.m. (**6**), 156.8 p.p.m. (**7**) and 142.0 p.p.m. (**8**). The broadening and down-field shift of ^113^Cd NMR peak in the induction period at 130 °C from 1 min (**5**) to 30 min (**7**) indicates the probable process of the formation of Cd—Te bonds. After nucleation/growth of CdTe NCs (**8**), the ^31^P and ^113^Cd NMR resonance signals become sharper and shift up-field.

We performed our reactions with a Cd to Te feed molar ratio of four to one and a Te concentration of 44 mmol kg^−1^ in oleylamine (OLA). For a typical reaction, the Cd precursor (1.54 ml, 0.88 mmol) was added to a three-neck reaction flask containing OLA (3.00 ml) in a glove box. Meanwhile, the Te precursor TOPTe (0.39 ml, 0.22 mmol Te) was diluted into OLA (1.50 ml) in a small vial. The total volume is about 6.44 ml. The experimental conditions regarding the Cd to Te feed molar ratio and the preparation of the Cd and Te precursors were the same for the study shown in Figs 2, 3, 4, 5. The flask and the vial were sealed and taken out of the glove box; the temperature of the Cd mixture was increased to 130 °C or 135 °C and the diluted Te precursor was transferred into the three-neck flask. As the reaction proceeded, aliquots of the reaction solution were taken at different intervals and kept in vials.

### Ultraviolet absorption spectroscopy

All of the ultraviolet absorption spectra were recorded on a Hitachi UH4150 spectrometer with a water circulation temperature controller. All the ultraviolet absorption spectra data were collected at the same sample concentration at room temperature (30 μl sample was diluted into 3 ml solvent), if not otherwise specified. For ultraviolet absorption study, Hellma fluorescence cuvettes were used (standard cells). The data collection interval is 1 nm and the scan rate is 800 nm·min^−1^. The light path was 10 mm (macro, suprasil quartz, limit 200–2,500 nm spectral range, path length 10 × 10 mm, chamber volume 3,500 μl). The background was corrected with the solvents used. Solvents for measurements at high temperatures were pre-heated before diluting the samples. It is of help to point out that the absorbance units are given as a.u. in our study; still, it is possible to compare the absorbance in a relative fashion for the various reactions presented in one figure, such as Fig. 2.

### Small-angle X-ray scattering

The SAXS measurements were conducted at BL16B1 beamline at Shanghai Synchrotron Radiation Facility (SSRF), Shanghai, China, using X-ray with the wavelength of *λ*=1.24 Å (i.e., with energy of 10 keV) as the incident beam. With the sample-to-detector distance of 1,887 mm, solution samples were injected into capillaries of 1.5 mm diameter as sample holders, which could be mounted on a Linkam heating stage (THMS 600) which allows the control of temperature.

The scattering intensity was recorded using a Rayonix SX-165 CCD detector (Rayonix, Evanston, IL, USA) with a resolution of 2,048 × 2,048 pixels and a pixel size of 80 μm × 80 μm. The scattering intensity *I*(*q*) is represented as a function of the modulus of the scattering vector *q*=(4π/*λ*)sin*θ*, where *λ* being the wavelength and 2*θ* being the scattering angle. All of the data were corrected for background and air scattering. The two-dimensional data were averaged to obtain the one dimensional SAXS profile by circular averaging with Fit2D software. *I*(*q*) was calibrated into absolute scale using glass carbon as standard reference.

The SAXS data were fitted with the unified model proposed by Beaucage^59^,^60^,^61^,^62^. The scattering intensity (in absolute scale) from each level of particles is given by a unified equation and the total intensity is summed from each level and can be fitted using the software package Irena as


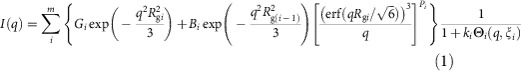


where *i* notes the *i*th structure level and *m* is the number of total levels. *G* is the classic Guinier prefactor. *R*_g_ is the radius of gyration. *B* is the Porod constant defined according to the regime in which the exponent *P* falls and erf is the error function. 1/[1+*k*Θ(*q*, *ξ*)] is the structure factor accounts for weak correlations between the particles in the same structure level with a correlation distance of *ξ* and a pack factor *k*, which describes the degree of correlation (0<*k*<5.92). Larger *k* corresponds to stronger interparticle scattering correlations and *k*=0 means no correlations. Θ for *i*th level is given by


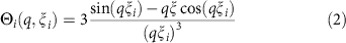


In each structure level, *ξ* describes the average Bragg-like spacing between the particles while the size and morphology of the particles can be determined by *R*_g_ and *P*. Generally^61^, for mass fractals *P*<3, while for surface fractals 3<*P*<4, and for diffuse interfaces *P*>4. More specifically, *P*=4 points to particles with smooth surfaces, *P*=3 points to very rough surfaces or ‘collapsed' polymer chains, *P*=2 can represent scattering either from Gaussian polymer chains or from a two-dimensional structure such as lamellae or platelets, *P*=5/3 points to scattering from ‘fully swollen' chains and *P*=1 represents scattering from a stiff rod^63^. *R*_g_ is related to both the particle shape and size, specially, the particle diameter *D* is given as 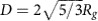 for spherical particle.

### Mass spectrometry

High-resolution electrospray mass spectrometry (ESI-MS) was performed in the positive ion mode on Agilent 6210A HPLC-TOF/MS. Agilent Mass Hunter software was used for analysing the data and operating the instrument. Pure acetonitrile was used as mobile phase. All samples were stored in dry toluene (with the 1–4 volume ratio of sample to toluene).

### Nuclear magnetic resonance spectroscopy

Nuclear magnetic resonance (NMR) samples were prepared in a glove box. A Bruker Avance III 400 MHz was used for ^31^P NMR and ^113^Cd NMR. 0.3 ml toluene-*d*_8_ was added to dilute the 0.3 ml sample to 0.6 ml. The P concentration was 0.081 mmol·ml^−1^ and the Cd concentration was 0.324 mmol·ml^−1^. For details, see Supplementary Table 3.

### Data availability

The authors declare that all relevant data supporting the findings of this study are available from the authors on request.

## Additional information

**How to cite this article:** Liu, M. *et al*. Probing Intermediates of the Induction period prior to nucleation and growth of semiconductor quantum dots. *Nat. Commun.* **8,** 15467 doi: 10.1038/ncomms15467 (2017).

**Publisher's note:** Springer Nature remains neutral with regard to jurisdictional claims in published maps and institutional affiliations.

## Supplementary Material

Supplementary Information Supplementary Figures, Supplementary Tables and Supplementary References.

## Figures and Tables

**Table 1:** Summary of our characterizations.

**Characterization**	**Intermediate 1 (RT)**	**Intermediate 2 (∼130** **°C)**
Ultraviolet (Fig. 2)	In Tol: ‘invisible'In amine-Tol: ‘invisible'	In Tol: ‘invisible'In amine-Tol: ‘visible' as MSC-371
SAXS (Fig. 3)	1.05 nm	0.97 nm (similar to that of MSC-371)
MS (Fig. 4)	No detection of Cd_*x*_Te_*y*_ fragments	7 to 12 atom structuredCd_*x*_Te_*y*_ fragments detected
NMR (Fig. 5)		
^31^P NMR: −27.2 p.p.m. (Te precursor)	down-field shift to −26.5 p.p.m.	up-field shift to −28.4 p.p.m.
^113^Cd NMR: 137.0 p.p.m. (Cd precursor)	137.2 p.p.m.	down-field shift and broadened to 156.8 p.p.m.

**Intermediate 1** formed at room temperature and **Intermediate 2** formed at elevated temperature (130 °C). Both intermediates are ‘optically-invisible' in conventional toluene, while **Intermediate 2** expresses as ‘optically-visible' MSCs when a primary amine is added. SAXS gives the size of ∼1 nm for the two intermediates. Cd_*x*_Te_*y*_ fragments were detected by MS from our **Intermediate 2** sample and not from **Intermediate 1** sample, suggesting the presence and absence of Cd—Te covalent bonds, respectively. NMR demonstrates the formation of the Cd—Te covalent bond. From the Cd/Te precursors, to **Intermediate 1**, and **Intermediate 2**, ^31^P/^113^Cd resonance signals were detected to shift from −27.2/137.0 p.p.m., to −26.5/137.2 p.p.m. (RT/24 h), and −28.4/156.8 p.p.m. (130 °C/30 min), respectively.

## References

[b1] Bruchez M., Moronne M., Gin P., Weiss S. & Alivisatos A. P. Semiconductor nanocrystals as fluorescent biological labels. Science 281, 2013–2016 (1998). 9748157 10.1126/science.281.5385.2013

[b2] Chan W. C. & Nie S. Quantum dot bioconjugates for ultrasensitive nonisotopic detection. Science 281, 2016–2018 (1998). 9748158 10.1126/science.281.5385.2016

[b3] Medintz I. L., Uyeda H. T., Goldman E. R. & Mattoussi H. Quantum dot bioconjugates for imaging, labelling and sensing. Nat. Mater. 4, 435–446 (2005). 15928695 10.1038/nmat1390

[b4] Lim S. J. . Brightness-equalized quantum dots. Nat. Commun. 6, 8210 (2015). 26437175 10.1038/ncomms9210 PMC4594210

[b5] Chuang C.-H. M., Brown P. R., Bulović V. & Bawendi M. G. Improved performance and stability in quantum dot solar cells through band alignment engineering. Nat. Mater. 13, 796–801 (2014). 24859641 10.1038/nmat3984 PMC4110173

[b6] Yuan M., Liu M. & Sargent E. H. Colloidal quantum dot solids for solution-processed solar cells. Nat. Energy 1, 16016 (2016).

[b7] Lewis N. S. Research opportunities to advance solar energy utilization. Science 351, aad1920 (2016). 26798020 10.1126/science.aad1920

[b8] Medintz I. L. . Self-assembled nanoscale biosensors based on quantum dot FRET donors. Nat. Mater. 2, 630–638 (2003). 12942071 10.1038/nmat961

[b9] Medintz I. L. . Quantum-dot/dopamine bioconjugates function as redox coupled assemblies for *in vitro* and intracellular pH sensing. Nat. Mater. 9, 676–684 (2010). 20651808 10.1038/nmat2811

[b10] Goldman E. R. . A hybrid quantum dot-antibody fragment fluorescence resonance energy transfer-based TNT sensor. J. Am. Chem. Soc. 127, 6744–6751 (2005). 15869297 10.1021/ja043677l

[b11] Ming K. . Integrated quantum dot barcode smartphone optical device for wireless multiplexed diagnosis of infected patients. ACS Nano 9, 3060–3074 (2015). 25661584 10.1021/nn5072792

[b12] Murray C., Norris D. J. & Bawendi M. G. Synthesis and characterization of nearly monodisperse CdE (E= sulfur, selenium, tellurium) semiconductor nanocrystallites. J. Am. Chem. Soc. 115, 8706–8715 (1993).

[b13] Peng Z. A. & Peng X. Nearly monodisperse and shape-controlled CdSe nanocrystals via alternative routes: nucleation and growth. J. Am. Chem. Soc. 124, 3343–3353 (2002). 11916419 10.1021/ja0173167

[b14] Yang Y. A., Wu H., Williams K. R. & Cao Y. C. Synthesis of CdSe and CdTe nanocrystals without precursor injection. Angew. Chem. Int. Ed. 44, 6712–6715 (2005). 10.1002/anie.200502279 16187382

[b15] Yu K. . Ultraviolet ZnSe_1−*x*_S_*x*_ gradient-alloyed nanocrystals via a noninjection approach. ACS Appl. Mater. Interfaces 4, 4302–4311 (2012). 22812274 10.1021/am3009828

[b16] Yu K. . Low-temperature approach to highly emissive copper indium sulfide colloidal nanocrystals and their bioimaging applications. ACS Appl. Mater. Interfaces 5, 2870–2880 (2013). 23486927 10.1021/am302951k

[b17] Hendricks M. P., Campos M. P., Cleveland G. T., Jen-La Plante I. & Owen J. S. A tunable library of substituted thiourea precursors to metal sulfide nanocrystals. Science 348, 1226–1230 (2015). 26068846 10.1126/science.aaa2951

[b18] Zhang J. . Bright gradient-alloyed CdSe_*x*_S_1–*x*_ quantum dots exhibiting cyan-blue emission. Chem. Mater. 28, 618–625 (2016).

[b19] van Embden J. & Mulvaney P. Nucleation and growth of CdSe nanocrystals in a binary ligand system. Langmuir 21, 10226–10233 (2005). 16229549 10.1021/la051081l

[b20] Liu H., Owen J. S. & Alivisatos A. P. Mechanistic study of precursor evolution in colloidal group II-VI semiconductor nanocrystal synthesis. J. Am. Chem. Soc. 129, 305–312 (2007). 17212409 10.1021/ja0656696

[b21] Rempel J. Y., Bawendi M. G. & Jensen K. F. Insights into the kinetics of semiconductor nanocrystal nucleation and growth. J. Am. Chem. Soc. 131, 4479–4489 (2009). 19275244 10.1021/ja809156t

[b22] Owen J. S., Chan E. M., Liu H. & Alivisatos A. P. Precursor conversion kinetics and the nucleation of cadmium selenide nanocrystals. J. Am. Chem. Soc. 132, 18206–18213 (2010). 21128655 10.1021/ja106777j

[b23] Yu K. . Effect of tertiary and secondary phosphines on low-temperature formation of quantum dots. Angew. Chem. Int. Ed. 52, 4823–4828 (2013). 10.1002/anie.201300568 23526710

[b24] Yu K. . The formation mechanism of binary semiconductor nanomaterials: shared by single-source and dual-source precursor approaches. Angew. Chem. Int. Ed. 52, 11034–11039 (2013). 10.1002/anie.201304958 24006135

[b25] Yu K. . Mechanistic study of the role of primary amines in precursor conversions to semiconductor nanocrystals at low temperature. Angew. Chem. Int. Ed. 53, 6898–6904 (2014). 10.1002/anie.201403714 24855040

[b26] Yu K. . General low-temperature reaction pathway from precursors to monomers before nucleation of compound semiconductor nanocrystals. Nat. Commun. 7, 12223 (2016). 27531507 10.1038/ncomms12223 PMC4992053

[b27] Steckel J. S., Yen B. K., Oertel D. C. & Bawendi M. G. On the mechanism of lead chalcogenide nanocrystal formation. J. Am. Chem. Soc. 128, 13032–13033 (2006). 17017765 10.1021/ja062626g

[b28] Evans C. M., Evans M. E. & Krauss T. D. Mysteries of TOPSe revealed: insights into quantum dot nucleation. J. Am. Chem. Soc. 132, 10973–10975 (2010). 20698646 10.1021/ja103805s PMC2924661

[b29] Empedocles S. A., Neuhauser R., Shimizu K. & Bawendi M. G. Photoluminescence from single semiconductor nanostructures. Adv. Mater. 11, 1243–1256 (1999).

[b30] Cui J. . Direct probe of spectral inhomogeneity reveals synthetic tunability of single-nanocrystal spectral linewidths. Nat. Chem. 5, 602–606 (2013). 23787751 10.1038/nchem.1654 PMC3843964

[b31] Wuister S. F., van Driel F. & Meijerink A. Luminescence and growth of CdTe quantum dots and clusters. Phys. Chem. Chem. Phys. 5, 1253–1258 (2003).

[b32] Dagtepe P., Chikan V., Jasinski J. & Leppert V. J. Quantized growth of CdTe quantum dots; observation of magic-sized CdTe quantum dots. J. Phys. Chem. C 111, 14977–14983 (2007).

[b33] Wang R. . Single-sized colloidal CdTe nanocrystals with strong bandgap photoluminescence. Chem. Commun. 8, 962–964 (2009). 10.1039/b818967f 19214330

[b34] Dukes A. D. III, McBride J. R. & Rosenthal S. J. Synthesis of magic-sized CdSe and CdTe nanocrystals with diisooctylphosphinic acid. Chem. Mater. 22, 6402–6408 (2010).

[b35] Kasuya A. . Ultra-stable nanoparticles of CdSe revealed from mass spectrometry. Nat. Mater. 3, 99–102 (2004). 14743211 10.1038/nmat1056

[b36] Kudera S. . Sequential growth of magic-size CdSe nanocrystals. Adv. Mater. 19, 548–552 (2007).

[b37] Ouyang J. . Multiple families of magic-sized CdSe nanocrystals with strong bandgap photoluminescence via noninjection one-pot syntheses. J. Phys. Chem. C 112, 13805–13811 (2008).

[b38] Jiang Z. J. & Kelley D. F. Role of magic-sized clusters in the synthesis of CdSe nanorods. ACS Nano 4, 1561–1572 (2010). 20192241 10.1021/nn100076f

[b39] Liu Y.-H., Wang F., Wang Y., Gibbons P. C. & Buhro W. E. Lamellar assembly of cadmium selenide nanoclusters into quantum belts. J. Am. Chem. Soc. 133, 17005–17013 (2011). 21905688 10.1021/ja206776g

[b40] Yu K. CdSe magic-sized nuclei, magic-sized nanoclusters and regular nanocrystals: monomer effects on nucleation and growth. Adv. Mater. 24, 1123–1132 (2012). 22432157 10.1002/adma.201104081

[b41] Wang Y. . Isolation of the magic-Size CdSe nanoclusters [(CdSe)_13_(n-octylamine)_13_] and [(CdSe)_13_(oleylamine)_13_]. Angew. Chem. Int. Ed. 51, 6154–6157 (2012). 10.1002/anie.201202380 PMC3540746 22581628

[b42] Beecher A. N. . Atomic structures and gram scale synthesis of three tetrahedral quantum dots. J. Am. Chem. Soc. 136, 10645–10653 (2014). 25003618 10.1021/ja503590h

[b43] Pan D., Ji X., An L. & Lu Y. Observation of nucleation and growth of CdS nanocrystals in a two-phase system. Chem. Mater. 20, 3560–3566 (2008).

[b44] Li M. . CdS magic-sized nanocrystals exhibiting bright band gap photoemission via thermodynamically driven formation. ACS Nano 3, 3832–3838 (2009). 19911809 10.1021/nn9009455

[b45] Wang R. . Homogeneously-alloyed CdTeSe single-sized nanocrystals with bandgap photoluminescence. J. Phys. Chem. C 113, 3402–3408 (2009).

[b46] Yu K., Ouyang J. & Leek D. M. *In-situ* observation of nucleation and growth of PbSe magic-sized nanoclusters and regular nanocrystals. Small 7, 2250–2262 (2011). 21735546 10.1002/smll.201100457

[b47] Gary D. C., Terban M. W., Billinge S. J. & Cossairt B. M. Two-step nucleation and growth of InP quantum dots via magic-sized cluster intermediates. Chem. Mater. 27, 1432–1441 (2015).

[b48] Muckel F. . Digital doping in magic-sized CdSe clusters. ACS Nano 10, 7135–7141 (2016). 27420556 10.1021/acsnano.6b03348

[b49] Gebauer D., Völkel A. & Cölfen H. Stable prenucleation calcium carbonate clusters. Science 322, 1819–1822 (2008). 19095936 10.1126/science.1164271

[b50] Vekilov P. G. Nucleation. Cryst. Growth Design 10, 5007–5019 (2010). 10.1021/cg1011633 PMC2995260 21132117

[b51] Demichelis R., Raiteri P., Gale J. D., Quigley D. & Gebauer D. Stable prenucleation mineral clusters are liquid-like ionic polymers. Nat. Commun. 2, 590 (2011). 22186886 10.1038/ncomms1604 PMC3247826

[b52] Dey A. . The role of prenucleation clusters in surface-induced calcium phosphate crystallization. Nat. Mater. 9, 1010–1014 (2010). 21076415 10.1038/nmat2900

[b53] Habraken W. J. . Ion-association complexes unite classical and non-classical theories for the biomimetic nucleation of calcium phosphate. Nat. Commun. 4, 1507 (2013). 23422675 10.1038/ncomms2490

[b54] Anwar J., Khan S. & Lindfors L. Secondary crystal nucleation: nuclei breeding factory uncovered. Angew. Chem. Int. Ed. 54, 14681–14684 (2015). 10.1002/anie.201501216 25809644

[b55] Nielsen M. H., Aloni S. & De Yoreo J. J. *In situ* TEM imaging of CaCO_3_ nucleation reveals coexistence of direct and indirect pathways. Science 345, 1158–1162 (2014). 25190792 10.1126/science.1254051

[b56] Smeets P., Cho K., Kempen R., Sommerdijk N. & De Yoreo J. Calcium carbonate nucleation driven by ion binding in a biomimetic matrix revealed by *in situ* electron microscopy. Nat. Mater. 14, 394–399 (2015). 25622001 10.1038/nmat4193

[b57] Leatherdale C. A. & Bawendi M. G. Observation of solvatochromism in CdSe colloidal quantum dots. Phys. Rev. B 63, 165315 (2001).

[b58] Li T., Senesi A. J. & Lee B. Small angle X-ray scattering for nanoparticle research. Chem. Rev. 116, 11128–11180 (2016). 27054962 10.1021/acs.chemrev.5b00690

[b59] Beaucage G., Kammler H. K. & Pratsinis S. E. Particle size distributions from small-angle scattering using global scattering functions. J. Appl. Cryst. 37, 523–535 (2001).

[b60] Beaucage G. Approximations leading to a unified exponential/power-law approach to small-angle scattering. J. Appl. Cryst. 28, 717–728 (1995).

[b61] Beaucage G., Ulibarri T. A., Black E. P. & Schaefer D. W. Multiple size scale structures in silica-siloxane composites studied by small-angle scattering. ACS Symposium Series 144American Chemical Society (1995).

[b62] Beaucage G. Small-angle scattering from polymeric mass fractals of arbitrary mass-fractal dimension. J. Appl. Cryst. 29, 134–146 (1996).

[b63] Hammouda B. A new Guinier–Porod model. J. Appl. Cryst. 43, 716–719 (2010).

[b64] Comeau A. N., Liu J., Khadka C. B., Corrigan J. F. & Konermann L. Nanocluster isotope distributions measured by electrospray time-of-flight mass spectrometry. Anal. Chem. 85, 1200–1207 (2013). 23214505 10.1021/ac3031674

[b65] Ratcliffe C. I. . Solid state NMR studies of photoluminescent cadmium chalcogenide nanoparticles. Phys. Chem. Chem. Phys. 8, 3510–3519 (2006). 16871340 10.1039/b606507b

[b66] Justino L. n. L. . Gel formation and interpolymer alkyl chain interactions with poly (9, 9-dioctylfluorene-2, 7-diyl) (PFO) in toluene solution: results from NMR, SANS, DFT, and semiempirical calculations and their implications for PFO β-phase Formation. Macromolecules 44, 334–343 (2010).

[b67] Jasieniak J. & Mulvaney P. From Cd-rich to Se-rich-the manipulation of CdSe nanocrystal surface stoichiometry. J. Am. Chem. Soc. 129, 2841–2848 (2007). 17309253 10.1021/ja066205a

[b68] Nam W. Synthetic mononuclear nonheme iron-oxygen intermediates. Acc. Chem. Res. 48, 2415–2423 (2015). 26203519 10.1021/acs.accounts.5b00218

[b69] Hoye T. R., Baire B., Niu D., Willoughby P. H. & Woods B. P. The hexadehydro-Diels-Alder reaction. Nature 490, 208–212 (2012). 23060191 10.1038/nature11518 PMC3538845

[b70] Battilocchio C. . Iterative reactions of transient boronic acids enable sequential C-C bond formation. Nat. Chem. 8, 360–367 (2016). 27001732 10.1038/nchem.2439

[b71] Garcia-Rodriguez R. & Liu H. Solution structure of cadmium carboxylate and its implications for the synthesis of cadmium chalcogenide nanocrystals. Chem. Commun. 49, 7857–7859 (2013). 10.1039/c3cc44103b 23900227

[b72] Garcia-Rodriguez R. & Liu H. A nuclear magnetic resonance study of the binding of trimethylphosphine selenide to cadmium oleate. J. Phys. Chem. A 118, 7314–7319 (2014). 24410663 10.1021/jp411681f

