# Supplementary material for: Probing intermediates of the induction period prior to nucleation and growth of semiconductor quantum dots
Source: Nat Commun. 2017 Jun 5;8:15467. doi: 10.1038/ncomms15467 (PMC5494182; doi:10.1038/ncomms15467)
Supplement: Supplementary Information — Supplementary Figures, Supplementary Tables and Supplementary References. [file ncomms15467-s1.pdf]

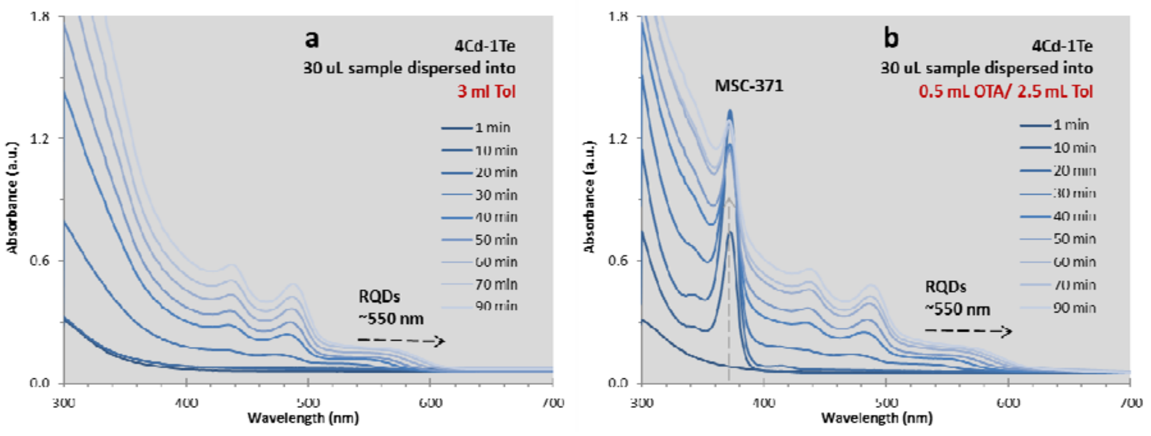

Supplementary Figure 1. The absorption spectra collected for the Fig. 2 batch. Nine (30 μL) samples were dispersed in 3 mL toluene (**a**) and in the mixture of 0.5 mL octylamine (OTA) and 2.5 mL toluene (**b**). The spectra were collected after 1 min of dispersion. The Cd and Te precursors were mixed at 135 °C for up to 90 min. The growth period was indicated for each of the nine samples. CdTe magic-size clusters (MSCs) which have absorption peaks at 490 nm (MSC-490) was reported before[1]. Significant efforts were carried out to explore the appropriate reaction condition including temperature, for the the induction period. For the formation of MSC-371 immediate intermediates under our experimental condition (such as with our Cd and Te precursors), the optimal formation temperature was found to be around ~130 °C.

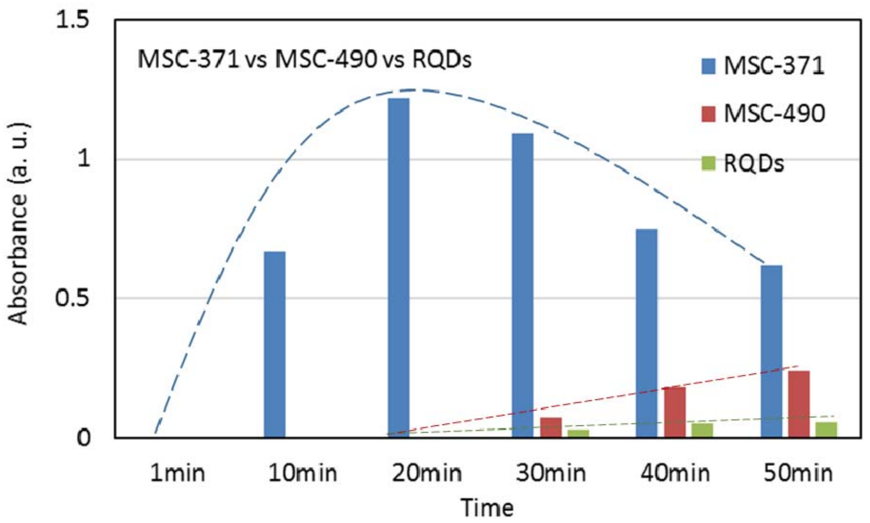

Supplementary Figure 2. The evolution of the nanocrystals (NCs) observed in Supplementary Fig. 1b. The *y* axis represents the absorption optical intensity obtained for the various NCs. The amount of MSC-371 seems to be affected by the nucleation/growth of regular quantum dots (RQDs).

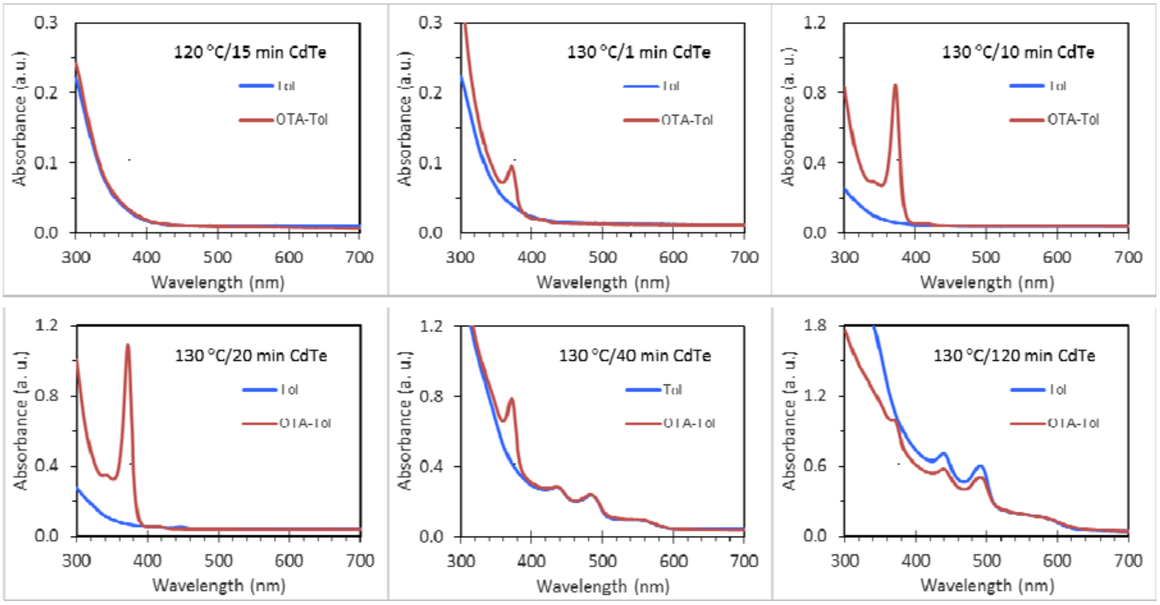

Supplementary Figure 3. The detection of MSC-371 from a different batch (other than Fig. 2 and Supplementary Fig. 1); the absorption spectra were collected after 1 min of sample dispersion. The same Cd and Te precursors with the same feed molar ratios were used but mixed at 120 °C. The reaction temperature and growth periods are indicated (which are 120 °C/15 min, 130 °C/1 min, 130 °C/10 min, 130 °C/20 min, 130 °C/40 min and 130 °C/120 min). A 30 µL sample was dispersed in 3 mL toluene (blue) or the mixture of 0.5 mL OTA/2.5 mL toluene (red).

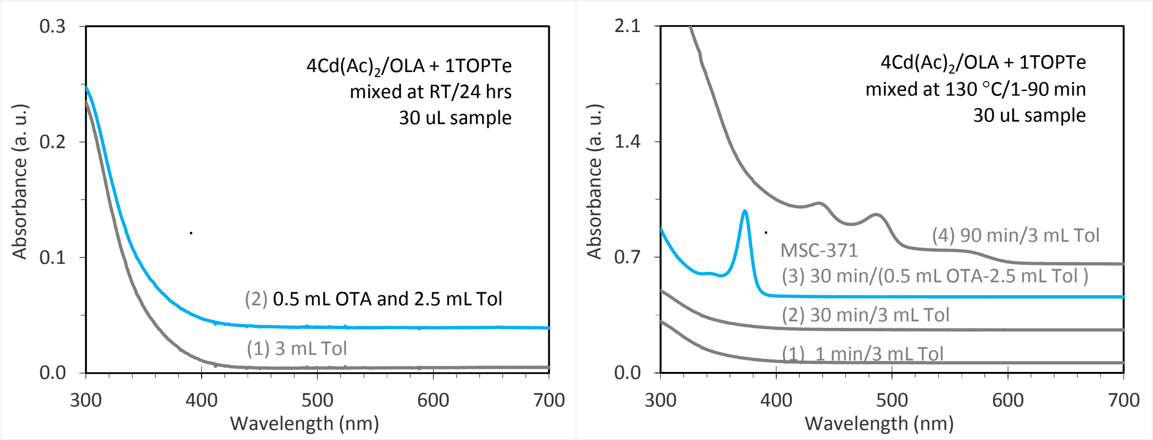

Supplementary Figure 4. Absorption spectra collected from the mixture of our Cd and Te precursors at room temperature (25 °C) for 24 h (**left**) and at 130 °C (**right**). For **left**, an aliquot of 30 μL sample (**Intermediate 1**) was dispersed in 3 mL toluene (1) and in the mixture of 0.5 mL OTA and 2.5 mL toluene (2, offset). For **right**, the growth periods were indicated; each spectrum was collected with an aliquot of 30 μL sample dispersed in 3 mL toluene (1, 2, and 4) or the mixture of 0.5 mL OTA and 2.5 mL toluene (3). The 130 °C/30 min sample is **Intermediate 2**. These experimental conditions were used to prepare our SAXS (Fig. 3), MS (Fig. 4) and NMR (Fig. 5) samples.

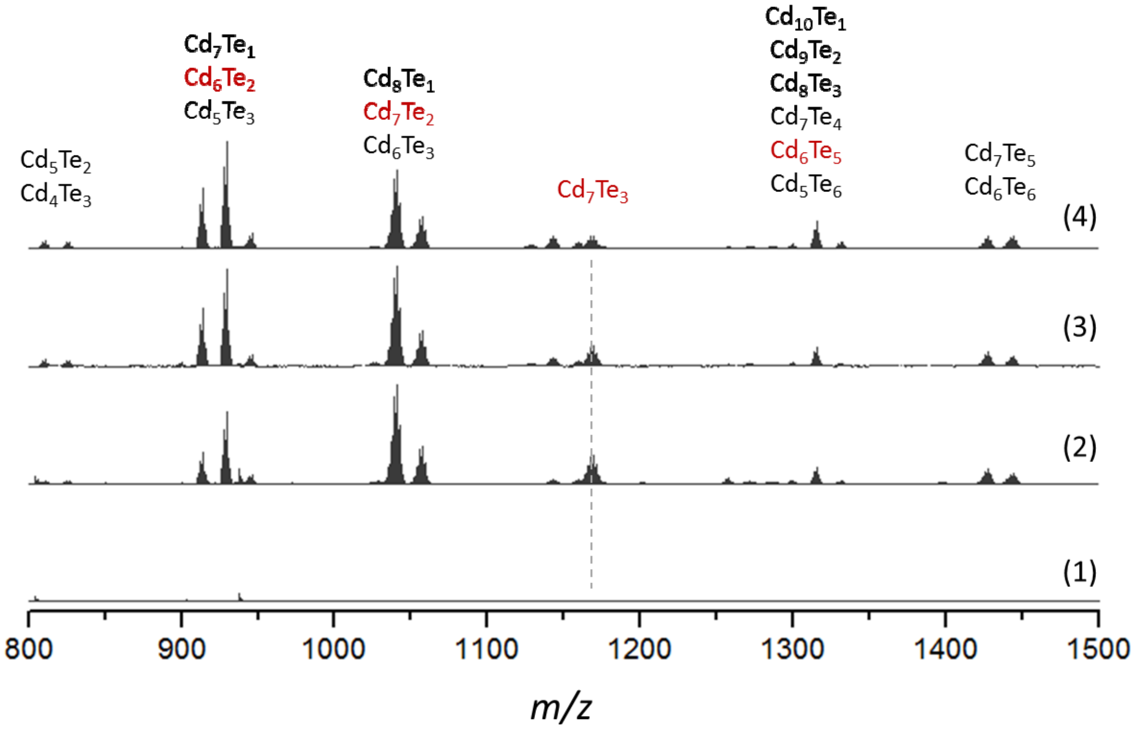

Supplementary Figure 5. ESI-Mass spectra in the region of *m/z* 800–1500 were collected from the RT/24 h sample **(1),** the 130 °C/1 min sample **(2),** the 130 °C/10 min sample **(3)** and the 130 °C/30 min sample **(4)**.

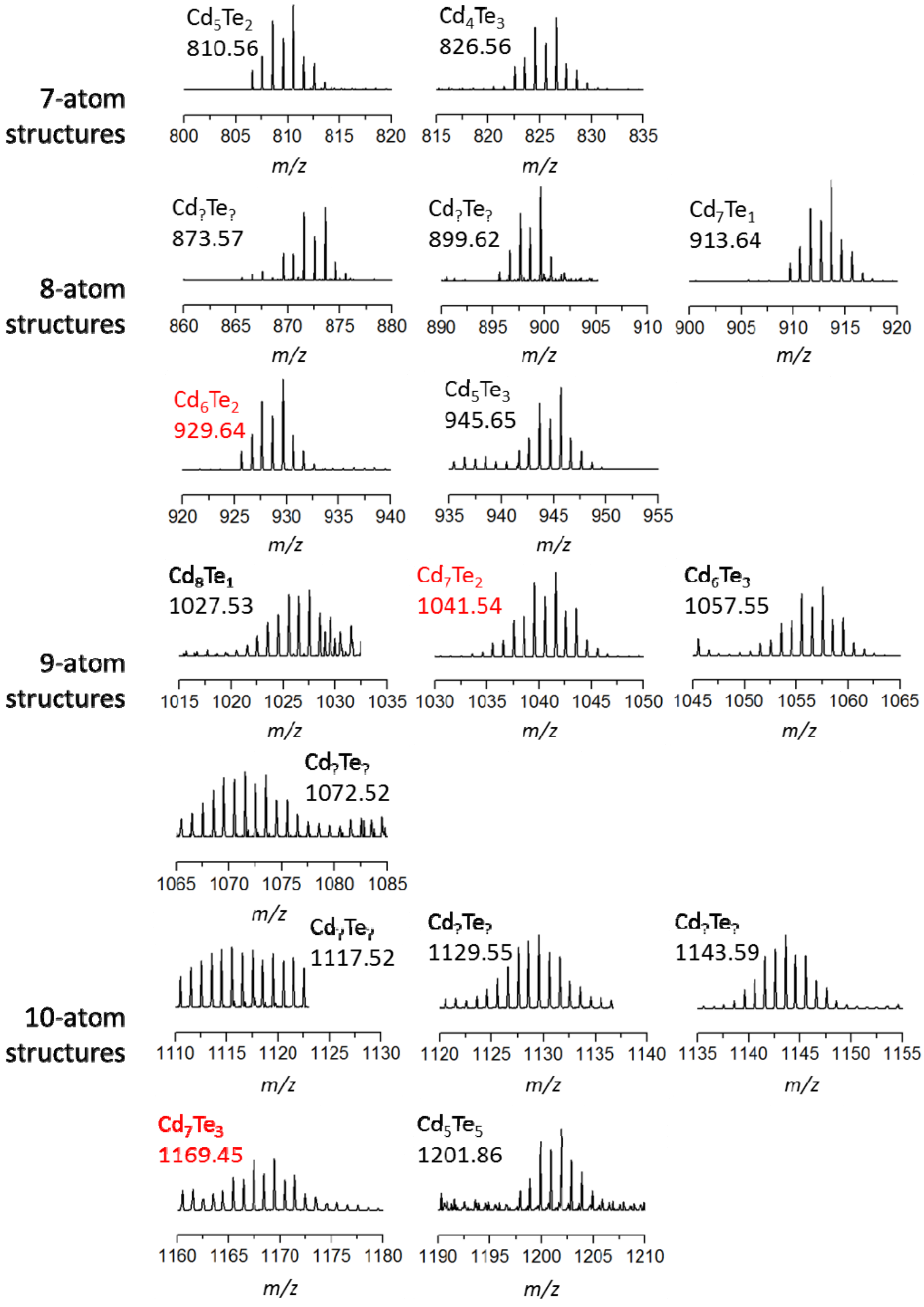

7-atom structures
Cd5Te2
810.56
Cd4Te3
826.56
8-atom structures
Cd?Te?
873.57
Cd?Te?
899.62
Cd7Te1
913.64
Cd6Te2
929.64
Cd5Te3
945.65
9-atom structures
Cd8Te1
1027.53
Cd7Te2
1041.54
Cd6Te3
1057.55
Cd?Te?
1072.52
10-atom structures
Cd?Te?
1117.52
Cd?Te?
1129.55
Cd?Te?
1143.59
Cd7Te3
1169.45
Cd5Te5
1201.86
m/z

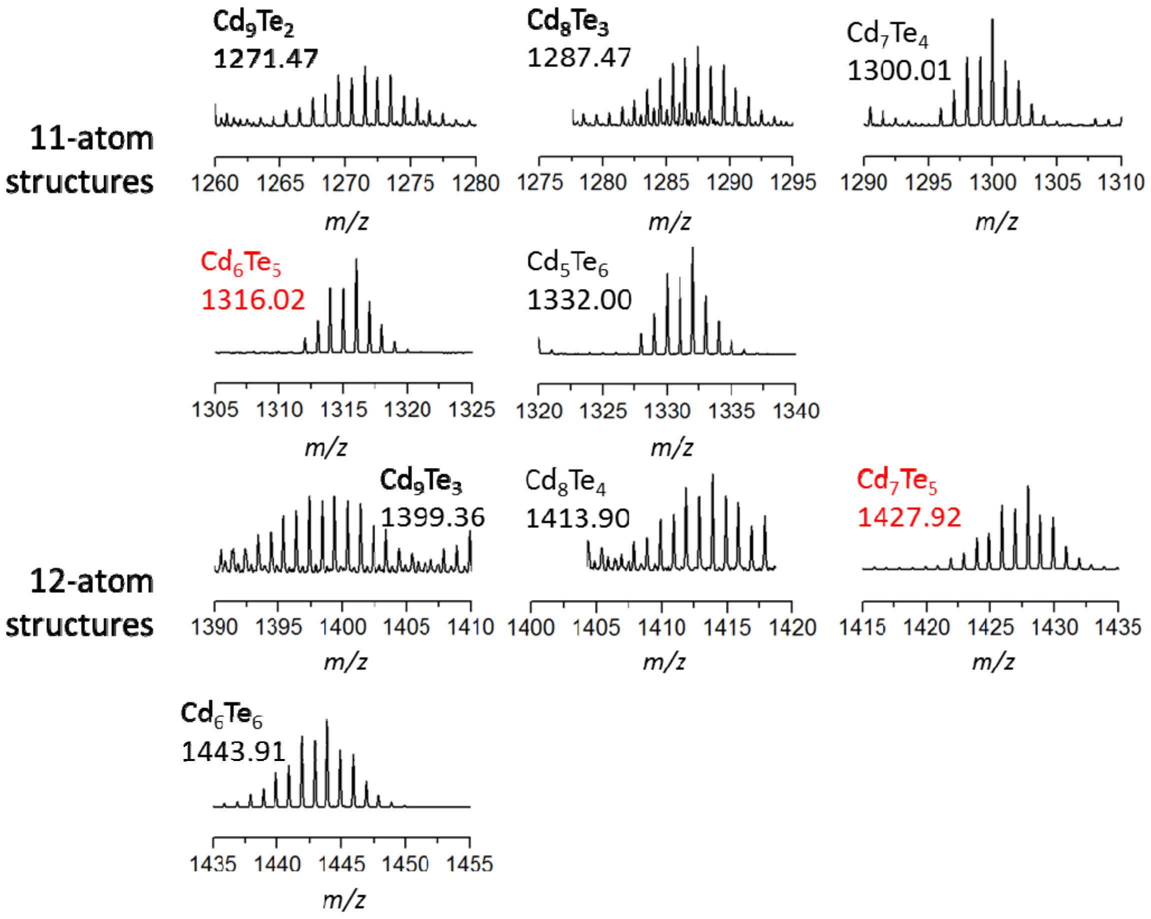

Supplementary Figure 6. The summary of the fragments detected from the 130 °C/30 min in the region of *m/z* 800–1500. The unmatchable fragments are labeled as $Cd_?Te_?$.

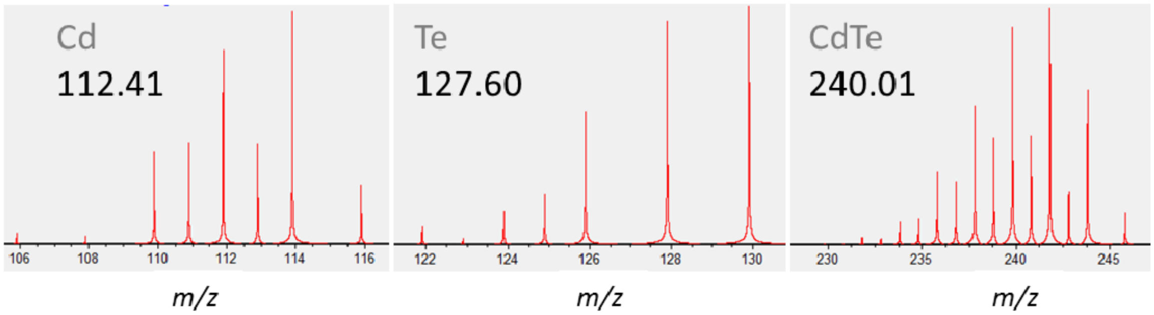

Supplementary Figure 7. The Cd (left) and Te (middle) isotope distribution from Isopro software with Lorentz fitting, together with the calculated CdTe (right) isotope distribution.

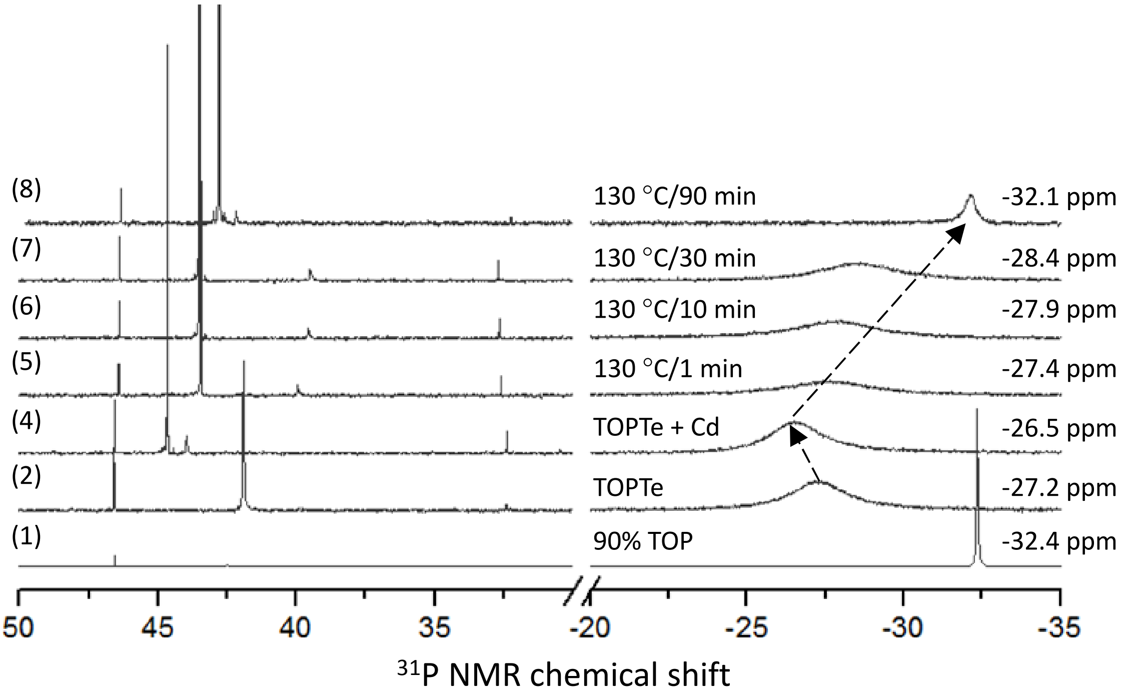

Supplementary Figure 8. ^{31}P {^{1}H} NMR investigation on the induction period of the reaction of $Cd(Ac)_2(OLA)_x$ + TOPTe (**4-8**), together with background experiments (**1-2**). Note that there are no nanocrystals formed even at 130 °C for 30 min (as detected in toluene with absorption spectroscopy shown in Supplementary Fig. 4b, spectrum 2). Interestingly, the chemical shift moving from -27.2 ppm (RT, **4**) to -27.4 ppm (130 °C/1 min, **5**), -27.9 ppm (130 °C/10 min, **6**), -28.4 ppm (130 °C/30 min, **7**) and to -32.1 ppm (130 °C/90 min, **8**) is coupled with the significant decrease of the ratio of the integral of this peak to all of the other P-containing species from 16.7 to 6.7, 5.8, 4.3 and to 0.8, respectively. Accordingly, the consumption of TOPTe is obvious from (**4-8**).

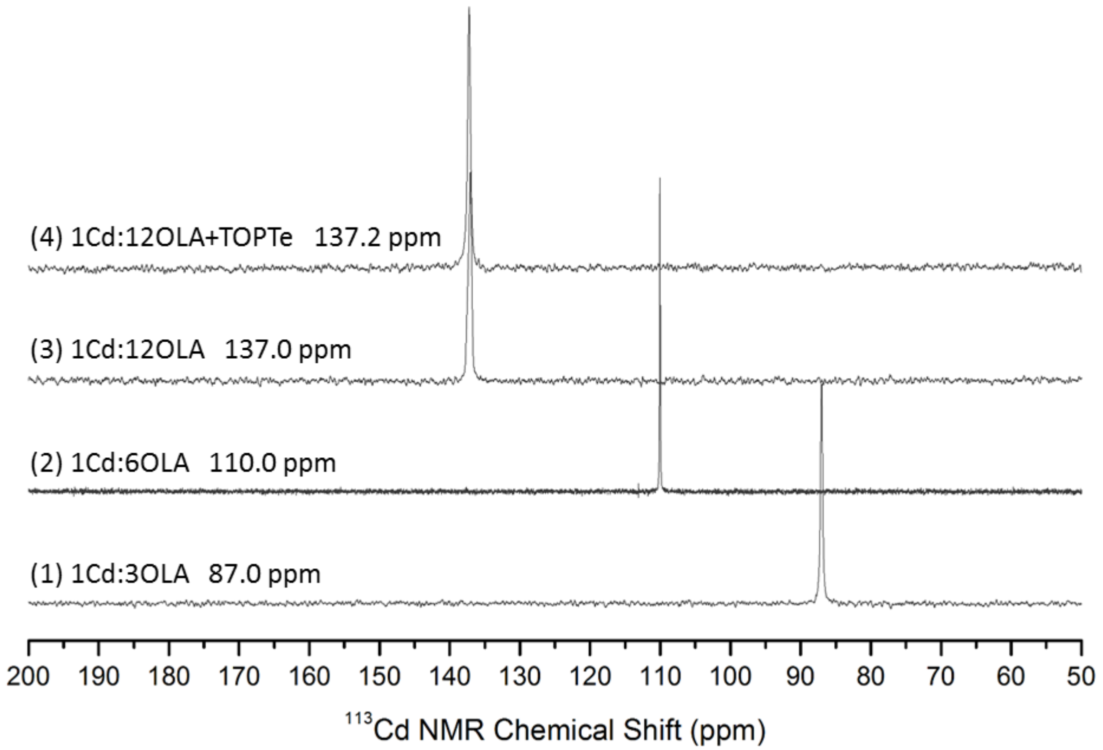

Supplementary Figure 9. The ^{113}Cd NMR spectra collected from four mixtures; $Cd(ClO_4)_2$ (0 ppm) was our reference used. $Cd(Ac)_2$ was dissolved into OLA and degassed at 120 °C for 30min, with the Cd to OLA feed molar ratios of 1 : 3 (**1**), 1 : 6 (**2**), and 1 : 12 (**3**). The 1Cd-to-12OLA mixture, our Cd precursor, was mixed with our Te precursor TOPTe with the feed molar ratio of 4 : 1 at RT for 24 hours (**4**). Before data collection, the four $Cd(Ac)_2$-containing mixtures were diluted into toluene-d_8 with the Cd concentration of 88 $mmol{\cdot}L^{-1}$ and the Te concentration of 22 $mmol{\cdot}L^{-1}$. For each of the four spectra, its collection took four hours with 4096 scans. See **Supplementary Table 1** for our summary on the literature study of ^{113}Cd NMR.

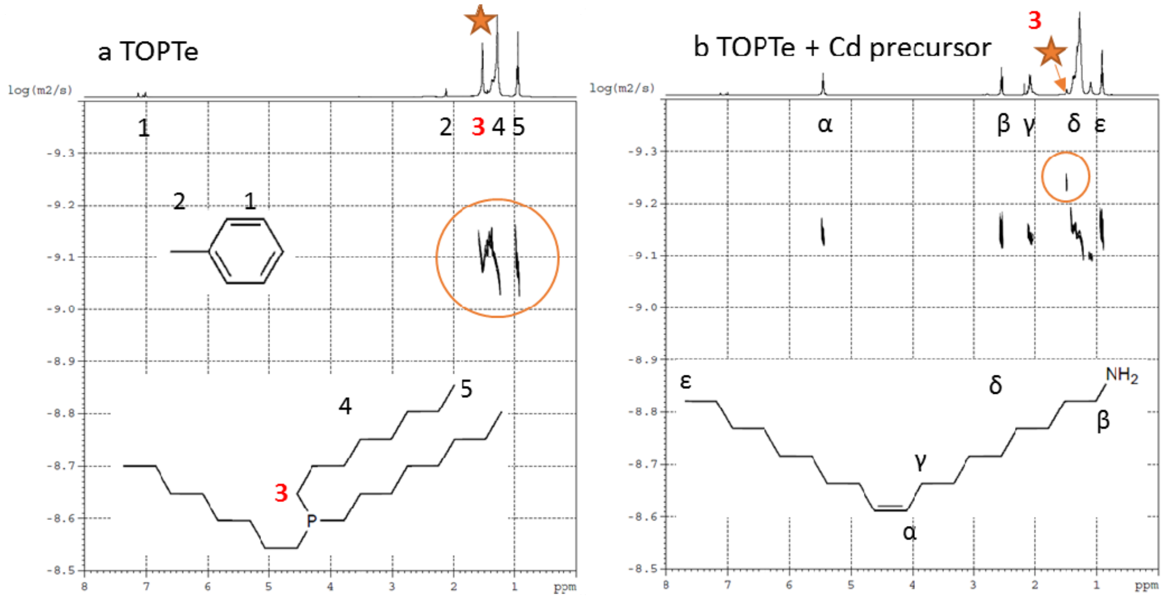

Supplementary Figure 10. Diffusion Ordered Spectroscopy (DOSY) was carried out for our Te precursor TOPTe **(a)** and the mixture of our Te and Cd precursors, TOPTe + Cd precursor, with the feed molar ratio of 1Te to 4Cd **(b)**. Toluene-d_8 was used. The diffusion coefficient (D, $m^2 \cdot s^{-1}$) was directly provided and was decreased from 8.50e-10 **(a**, y axis = -9.07**)** to 5.85e-10 **(b,** y axis = -9.23**)**. The y axis is log D ($m^2 \cdot s^{-1}$). The signal was corrected with $GdCl_3$ (0.1 $mg \cdot mL^{-1}$) in D_2O (1% H_2O). The ^1H resonance at 1.48 ppm (labeled by stars) is the α-protons near the P atom of TOPTe; interestingly, only this peak of TOPTe was detected in **b**, and the other protons of TOPTe were not detected.

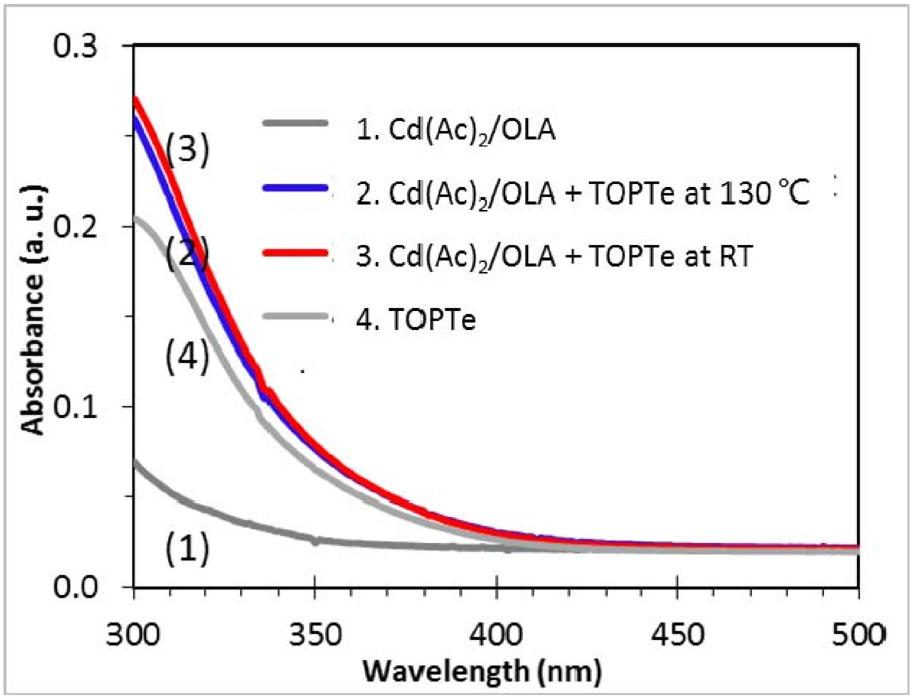

Supplementary Figure 11. The absorption spectra collected in 3 mL toluene, with 30 µL Cd precursor in OLA (consisting of 9 µL Cd precursor with 0.004 mmol Cd, 1), a 30 µL sample of the mixture of the Cd and Te precursors at 130 °C for 30 min (2, **Intermediate 2**), another 30 µL sample at room temperature for 24 h (3, **Intermediate 1**), and 30 µL Te precursor in OLA (consisting of 1 µL TeTOP precursor with 0.001 mmol Te, 4). Importantly, the concentrations of Cd and Te in the four dispersions were adjusted to be similar, regarding the total volume of 6.44 mL of the Cd and Te precursors used for our reactions. It is worthy of notice that Spectrum 2 and Spectrum 3 are very much similar, and there was little nucleation and growth for the two experimental conditions.

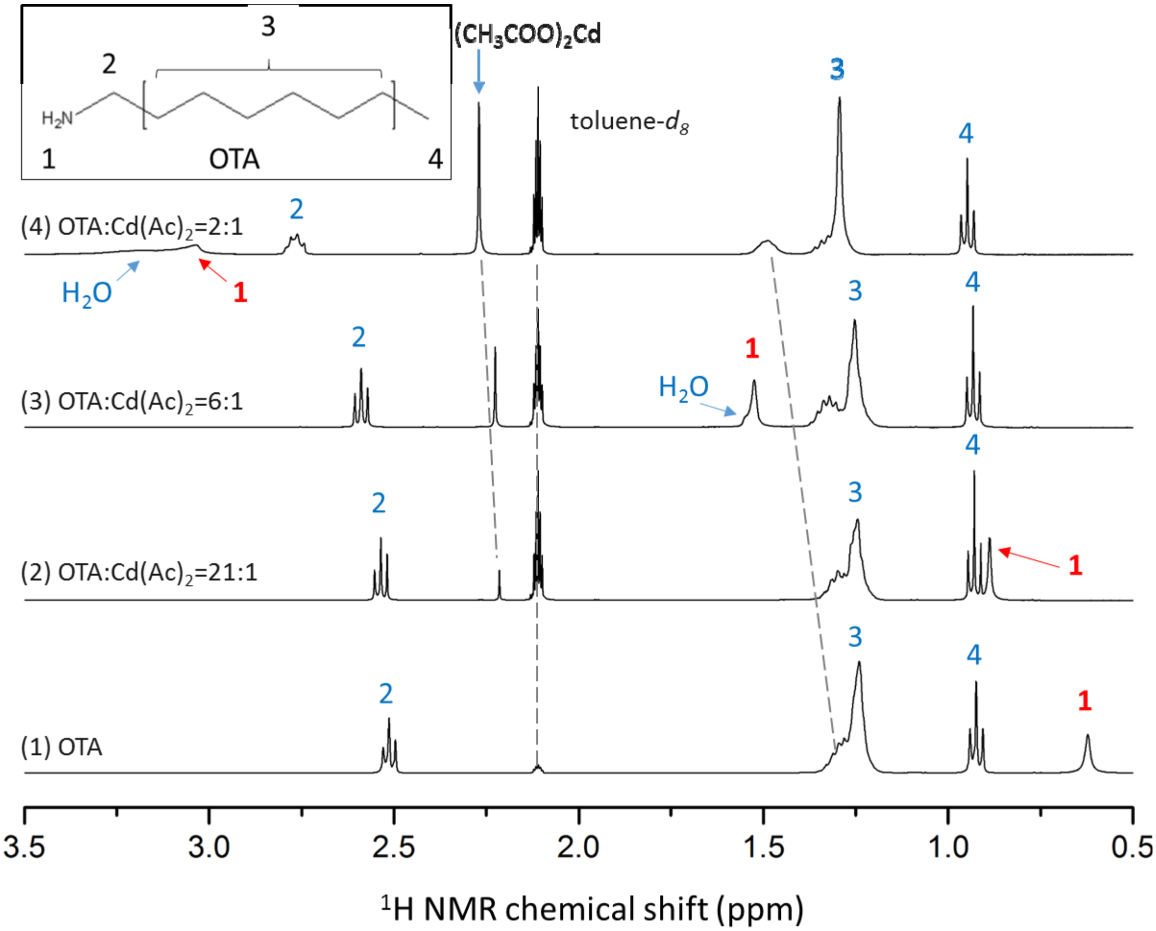

Supplementary Figure 12. ^{1}H NMR spectra of OTA (commercial) **(1),** the mixtures of OTA and our Cd source $Cd(OAc)_2{\cdot}2H_2O$ with the feed molar ratios of 21 : 1 **(2)**, 6 : 1 **(3)** and 2 : 1 **(4)**. The mixtures were prepared by the addition of OTA to toluene-d_8; afterwards, the Cd source was added. The OTA concentrations in toluene were about 20 mg OTA in 1 mL toluene (1) and 2 mg OTA in 1 mL (2-4). Our assignments of the peaks are mainly based on integral. It is obviously that the chemical shift of the very proton (labeled as number **1**, traces 2-4) bonded to the nitrogen atom of the OTA molecule in the mixtures with the Cd source is different from that of a free OTA (trace 1, 0.63 ppm). Thus, to induce the presence of MSC-371 from **Intermediate 2** prepared in Fig. 2 and Supplementary Figs. 1 and 3, the addition of primary amine molecules into toluene was found to be necessary.

Supplementary Table 1. Summary of the literature study on ^{113}Cd NMR[2-5].

Materials	Solvents	Chemical Shift	Standard
bulk CdTe	solid	395	$Cd(NO_3)_2{\cdot}4H_2O$ [2]
CdTe RQDs	solid	372	
Core of CdTe MSC-428	solid	485	$Cd(NO_3)_2{\cdot}4H_2O$ [3]
Surface of CdTe MSC-428	solid	357	
$Cd(NO_3)_2{\cdot}4H_2O$	solid	-100	$Cd(ClO_4)_2$ in D_2O [4]
$Cd(OAc)_2{\cdot}2H_2O$	solid	-46	
Cadmium Oleate	toluene	-9.1	$Cd(ClO_4)_2$ in D_2O [5]
$Cd(OAc)_2{\cdot}2H_2O$ in OTA	DMSO	155	

Supplementary Table 2. Fitted parameters and errors of SAXS profiles. Samples at 30 °C up to 150 °C and 130 °C/30 min have the scattering profile of a single level (L1), in which m = 1 and B = 0. The parameter P in L1 cannot be subtracted from the q range measured. Samples of MSCs have the scattering pattern of level 2 (L2), in which m = 2, i = 1 corresponds to L1 while i = 2 corresponds to L2 and $k_1 = k_2 = 0$.

Samples	R_g/Å	*error*/Å	*D*/Å	*error*/Å	*k*	*error*	*ξ*/Å	*error*/Å	R_g/Å	*error*/Å	*D*/Å	*error*/Å	*P*	*error*
30 °C	4.06	0.01	10.47	0.04	2.06	0.02	23.18	0.03						
40 °C	4.54	0.02	11.71	0.04	2.40	0.03	23.11	0.03						
50 °C	4.41	0.02	11.38	0.05	2.07	0.03	23.16	0.03						
60 °C	4.64	0.02	11.98	0.06	2.15	0.03	23.17	0.03						
70 °C	4.65	0.01	12.01	0.02	2.11	0.01	23.42	0.03						
80 °C	4.64	0.03	11.98	0.08	1.73	0.03	23.10	0.04						
90 °C	4.95	0.03	12.77	0.09	1.80	0.03	22.79	0.04						
100 °C	4.92	0.01	12.70	0.03	1.77	0.01	23.24	0.04						
110 °C	5.54	0.05	14.29	0.13	1.82	0.04	22.34	0.05						
120 °C	5.71	0.01	14.72	0.04	2.09	0.01	23.07	0.05						
130 °C	5.97	0.06	15.41	0.15	1.79	0.04	21.96	0.05						
140 °C	5.78	0.06	14.93	0.16	1.57	0.04	22.05	0.06						
150 °C	5.80	0.07	14.97	0.18	1.39	0.04	21.68	0.07						
130 °C/30 min	3.76	0.05	9.69	0.14	0.59	0.02	28.19	0.06						
MSC-371	3.74	0.01	9.66	0.03					75.50	1.27	194.79	3.26	2.60	0.11
MSC-417	5.39	0.20	13.89	0.52					84.36	2.27	217.66	5.85	1.40	0.06
MSC-448	6.41	0.02	16.53	0.05					93.94	2.40	242.37	6.19	2.10	0.06

Supplementary Table 3. Parameters of ^{113}Cd and ^{31}P NMR measurements.

**Nucleus**	**^{113}Cd**	**^{31}P NMR**
**Solvent**	Toluene-d_8	Toluene-d_8
**Temperature**	300.2 K	293.8 K
**Pulse Sequence**	zgig30	zgpg30
**Number of Scans**	4096	128
**Receiver Gain**	41	204
**Relaxation Delay**	3.0000	2.0000
**Pulse Width**	10.1300	7.8000
**Spectrometer Frequency**	88.80	161.98
**Standards**	H_3PO_4	$Cd(ClO_4)_2$

Supplementary References

1. Wang, Y., Zhou, Y., Zhang, Y. & Buhro, W. E. Magic-size II–VI nanoclusters as synthons for flat colloidal nanocrystals. *Inorg. Chem.* **54**, 1165-1177 (2015).
2. Ratcliffe CI, Yu K, Ripmeester JA, Badruz Zaman M, Badarau C & Singh S. Solid state NMR studies of photoluminescent cadmium chalcogenide nanoparticles. *Phys. Chem. Chem. Phys.* **8**, 3510-3519 (2006).
3. Wang R, et al. Homogeneously-alloyed CdTeSe single-sized nanocrystals with bandgap photoluminescence. *J. Phys Chem. C* **113**, 3402-3408 (2009).
4. Mennitt PG, Shatlock MP, Bartuska VJ & Maciel GE. Cadmium-113 NMR studies of solid cadmium(II) complexes. *J. Phys.Chem.* **85**, 2087-2091 (1981).
5. Garcíarodríguez R, Liu H. Mechanistic insights into the role of alkylamine in the synthesis of CdSe nanocrystals. *J. Am. Chem. Soc.* **136**, 1968-1975 (2014).
